# New Imidazolium Alkaloids with Broad Spectrum of Action from the Marine Bacterium *Shewanella aquimarina*

**DOI:** 10.3390/pharmaceutics15082139

**Published:** 2023-08-14

**Authors:** Rosa Giugliano, Gerardo Della Sala, Carmine Buonocore, Carla Zannella, Pietro Tedesco, Fortunato Palma Esposito, Costanza Ragozzino, Annalisa Chianese, Maria Vittoria Morone, Valerio Mazzella, Laura Núñez-Pons, Veronica Folliero, Gianluigi Franci, Anna De Filippis, Massimiliano Galdiero, Donatella de Pascale

**Affiliations:** 1 Department of Experimental Medicine, University of Campania “Luigi Vanvitelli”, 80138 Naples, Italy; rosa.giugliano@unicampania.it (R.G.); carla.zannella@unicampania.it (C.Z.); annalisa.chianese@unicampania.it (A.C.); mariavittoria.morone@unicampania.it (M.V.M.); anna.defilippis@unicampania.it (A.D.F.); 2 Department of Ecosustainable Marine Biotechnology, Stazione Zoologica Anton Dohrn, Via Ammiraglio Acton, 55, 80133 Naples, Italy; gerardo.dellasala@szn.it (G.D.S.); carmine.buonocore@szn.it (C.B.); pietro.tedesco@szn.it (P.T.); fortunato.palmaesposito@szn.it (F.P.E.); costanza.ragozzino@studenti.unime.it (C.R.); 3 Department of Chemical, Biological, Pharmaceutical and Environmental Sciences, University of Messina, Viale F. Stagno d’Alcontres, 31, 98166 Messina, Italy; 4 Department of Integrative Marine Ecology (EMI), Stazione Zoologica Anton Dohrn, Ischia Marine Centre, Ischia, 80077 Naples, Italy; valerio.mazzella@szn.it; 5 NBFC, National Biodiversity Future Center, 90133 Palermo, Italy; laura.nunezpons@szn.it; 6 Department of Integrative Marine Ecology (EMI), Stazione Zoologica Anton Dohrn, Villa Comunale, 80121 Napoli, Italy; 7 Department of Medicine, Surgery and Dentistry “Scuola Medica Salernitana”, University of Salerno, 84081 Baronissi, Italy; vfolliero@unisa.it (V.F.); gfranci@unisa.it (G.F.)

**Keywords:** *Shewanella*, imidazolium alkaloids, mass spectrometry, *S. aureus*, biosurfactant, antibiofilm, poliovirus, herpesvirus, coronavirus, *C. elegans*

## Abstract

The continuous outbreak of drug-resistant bacterial and viral infections imposes the need to search for new drug candidates. Natural products from marine bacteria still inspire the design of pharmaceuticals. Indeed, marine bacteria have unique metabolic flexibility to inhabit each ecological niche, thus expanding their biosynthetic ability to assemble unprecedented molecules. The One-Strain-Many-Compounds approach and tandem mass spectrometry allowed the discovery of a *Shewanella aquimarina* strain as a source of novel imidazolium alkaloids via molecular networking. The alkaloid mixture was shown to exert bioactivities such as: (a) antibacterial activity against antibiotic-resistant *Staphylococcus aureus* clinical isolates at 100 µg/mL, (b) synergistic effects with tigecycline and linezolid, (c) restoration of MRSA sensitivity to fosfomycin, and (d) interference with the biofilm formation of *S. aureus* 6538 and MRSA. Moreover, the mixture showed antiviral activity against viruses with and without envelopes. Indeed, it inhibited the entry of coronavirus HcoV-229E and herpes simplex viruses into human cells and inactivated poliovirus PV-1 in post-infection assay at 200 µg/mL. Finally, at the same concentration, the fraction showed anthelminthic activity against *Caenorhabditis elegans*, causing 99% mortality after 48 h. The broad-spectrum activities of these compounds are partially due to their biosurfactant behavior and make them promising candidates for breaking down drug-resistant infectious diseases.

## 1. Introduction

Infectious diseases caused by pathogens such as bacteria, fungi, and viruses pose a critical threat to public health. The Institute for Health Metrics and Evaluation (IHME) estimates more than 480 million of years of life were lost due to infectious diseases in 2019 [1]. A prominent cause is the ability of drug-resistant microorganisms to spread widely, which can provoke epidemics and pandemics due to antimicrobial resistance. Gram-positive bacteria, such as the methicillin-resistant *Staphylococcus aureus* (MRSA), often cause serious health care problems and community-associated infections [2]. *S. aureus* represents the major cause of respiratory tract and surgical site infections and the second source of bacteremia and device-related infections, causing the development of severe clinical complications, due to its great ability to acquire resistance and form biofilms [3,4]. Viruses also greatly contribute to the global burden of infectious diseases. Among the viral pathogens, enveloped viruses are responsible for common hospital and community diseases [5]. The latest available estimates report that herpetic infections due to herpes simplex virus type 1 (HSV-1) and herpes simplex virus type 2 (HSV-2) affect, respectively, 67 and 13% of people under 50 [6]. Unfortunately, resistance to antivirals often develops in immunocompromised patients, leading to proliferation of infection that worsens the clinical picture [7]. Due to horizontal transmission, viruses are very contagious and responsible for numerous pandemics. The latest originated from the etiological agent Severe Acute Respiratory Syndrome coronavirus 2 (SARS-CoV-2) in 2019 and caused millions of victims and burdened the world economic system. SARS-CoV-2 is a member of the *Coronaviridae* family that includes Human coronaviruses (HCoVs) HCoV-229E, NL63, OC43, and HKU1, responsible for approximately 30% of colds worldwide but also for severe and life-threatening infections in infants or immunocompromised people [8]. The high prevalence of antimicrobial resistance and the reduced development of new antimicrobial drugs underline the urgent need for new pharmaceuticals. The marine habitat represents a rich, unexplored source of bioactive compounds of various chemical natures which could be used as a basis to design new antimicrobial drugs [9]. The oceans cover about 70% of the Earth, and are characterized by wide variations in pressure, light, and temperature that influence the chemical, physical, and hydrological features of each specific habitat harboring many vital species [10,11]. The enormous marine biodiversity is due to the molecular diversity of the unique compounds produced by marine animals, plants, and microbes to adapt to the challenging conditions of their ecosystem. Many of these compounds are associated with different biological activities [12,13,14,15]. For example, marine biosurfactants, amphiphilic molecules able to lower the surface tension, have a wide range of biological and biotechnological activities, such as antitumor, antibacterial, antifungal, antiviral, and antioxidant [16,17,18].

More than 20,000 natural marine products of potential biotechnological relevance have been identified in the last 50 years [19]. The most frequent sources of antimicrobial compounds are represented by bacteria belonging to the phyla Actinobacteria, Bacteroidetes, Cyanobacteria, Firmicutes, Planctomycetes, and Proteobacteria, widely distributed in the marine environment [20]. In this work we focused our attention on the bacterium *Shewanella aquimarina* belonging to the phylum Proteobacteria and the class of Gamma-proteobacteria. Members of this genus are rod-shaped and facultative anaerobic Gram-negative bacteria, isolated from different marine and aquatic environments, such as fresh deep-water, ocean sediments, and sea ice. Their ability to survive in different aquatic environments is due to their remarkable physiological adaptability, and the considerable variability of bioactive metabolites released into the surrounding environment [21].

Herein we evaluated the different biological activities of a suite of secondary metabolites from the marine bacterium *Shewanella aquimarina*. In particular, the bacterium turned out to be a factory of imidazole alkaloids, most of them described here for the first time. Alkaloids are a broad and varied category of compounds that have provided the structural backbone for designing various antibiotics with a different range of action [22]. In addition, imidazole alkaloids have been reported as potential drugs for the treatment of tumors and cardiovascular diseases [23,24]. Although many marine-derived imidazole alkaloids come from sponges, recently such molecules have been isolated from marine bacteria and reported to possess several biological activities, including antibacterial and anthelmintic [25,26,27,28,29,30]. Therefore, to identify novel antimicrobial drug lead compounds, secondary metabolites from *Shewanella aquimarina* were structurally characterized through high-resolution mass spectrometry and, successively, their antibacterial, antiviral, and anthelmintic activities were assessed. The broad-spectrum activities of these compounds place them among the potential solutions for breaking down drug-resistant infectious diseases.

## 2. Materials and Methods

### 2.1. Chemicals, Media, and Buffers

Media utilized for *S. aquimarina* extract production

- LB broth (Lennox) (Condalab, Madrid, Spain);
- Marine Broth (MB) (Condalab, Madrid, Spain);
- Trypticasein Soy Broth (TSB) (Condalab, Madrid, Spain);
- Thioglycollate Medium ISO (TGB) (Condalab, Madrid, Spain);
- ISP2 (glucose 4 g/L, YE 4 g/L, malt extract 10 g/L);
- AUR (starch 10 g/L, glucose 10 g/L, glycerol 10 g/L, peptone 5 g/L, and YE 2 g/L);
- Mineral Salt Medium (MSM) (KH_2_PO_4_ 0.7 g/L, Na_2_HPO_4_ 0.9 g/L, NaNO_3_ 2 g/L, MgSO_4_ 0.4 g/L, and CaCl_2_ 0.1 g/L) supplemented with:
- glucose 10 g/L (MSM Glu);
- glucose 10 g/L and arginine 30 mM (MSM Glu Arg).

Media and buffer utilized for bacterial pathogen maintenance and testing

- Mueller Hinton (MH) broth (Merck KgaA, Darmstadt, Germany);
- MH supplemented with 1% (*w*/*v*) glucose (MHG);
- Dulbecco’s phosphate buffered saline (DPBS) (Microgem, Naples, Italy).

Medium utilized for mammalian cells and viruses

Dulbecco’s Modified Eagle Medium (DMEM) supplemented with 100 IU/mL penicillin-streptomycin solution and 10% (*v*/*v*) Fetal Bovine Serum (FBS) (Microgem, Naples, Italy).

Medium utilized for nematodes

Nematode Growth Medium (NGM) (2.5 g/L Peptone, 2.9 g/L NaCl, 17 g/L Bacto-Agar, 1 mM CaCl_2_, 5 µg/mL Cholesterol, 25 mM KH_2_PO_4_, and 1 mM MgSO_4_.

Antibiotics

The following antibiotics loaded on disks were utilized in the Kirby–Bauer assay: moxifloxacin (MFX) 5 µg, fosfomycin (FOT) 200 µg, erythromycin (E) 15 µg, linezolid (LZD) 10 µg, 30 µg, and tigecycline (TGC) 15 µg, purchased from Mast Group Ltd. (Bootle, UK), while ampicillin (AMP) 10 µg, cefepime (FEP) 30 µg, teicoplanin (TEC), and gentamycin (CN) 15 µg were bought from Oxoid Ltd. (Thermo Fisher Scientific, Waltham, MA, USA).

Acyclovir and Pleconaril utilized in the antiviral assays were obtained from MerK (Merck KgaA, Darmstadt, Germany).

Solvents

Dimethyl sulfoxide (DMSO) and 3-(4,5-dimethylthiazol-2-yl)-2,5-diphenyl tetrazolium bromide (MTT) were obtained from Sigma-Aldrich (Sigma-Aldrich, St. Louis, MO, USA). Ethyl acetate (EtOAc) and methanol (MeOH) utilized for the extractions and fractionation were obtained from ROMIL (ROMIL Ltd., Cambridge, UK). Mass-grade MeOH utilized for mass spectrometry was bought from CARLO ERBA (CARLO ERBA Reagents, Cornaredo, Italy).

### 2.2. Propagation and Maintenance of Biological Specimens

*S. aquimarina* CIP 108633T was obtained from the Pasteur Institute Collection (CIP), stocked in 20% (*v*/*v*) glycerol at −80 °C and routinely propagated on TYP agar at 20 °C.

A panel of both Gram-positive and Gram-negative bacterial pathogens, stored at −80 °C and routinely propagated on Mueller Hinton agar, were utilized as follows: *Staphylococcus aureus* ATCC 6538, *Staphylococcus epidermidis* ATCC 13360, *Enterococcus faecalis* ATCC 29212, *Escherichia coli* ATCC 25992, *Klebsiella pneumoniae* ATCC 10031, *Pseudomonas aeruginosa* O1, and *Salmonella enterica* serovar Typhimurium ATCC 14028. Five clinical isolates of *S. aureus* were also utilized. These strains were collected and characterized at the Laboratory of Microbiology of the “Luigi Vanvitelli” University Hospital using a BD Phoenix M50 instrument (Becton, Dickinson and Company, Franklin Lakes, NJ, USA). Their origin and drug resistance are reported in the following table (Table 1) [31].

#### 2.2.1. Cell Line and Viruses

Vero ATCC CCL-81 cells (ATCC, Manassas, VA, USA) were used for antiviral assays as susceptible to viral infection. The cell line was maintained in DMEM in a humidified atmosphere with 5% CO_2_ at 37 °C.

The viruses utilized in this work are herpes simplex virus type-1 strain SC16 (HSV-1), herpes simplex virus type-2 strain 333 (HSV-2), a strain of herpes acyclovir-resistant (HSV-1r) obtained by propagating HSV-1 on Vero cells with suboptimal ACV (Sigma-Aldrich) treatment, and Human coronavirus strain 229E ATCC VR-740 (HCoV-229E), Poliovirus Type 1 (PV-1) strain Chat ATCC VR-1562. All viral strains were propagated on a Vero cell monolayer and were stored at −80 °C.

#### 2.2.2. Caenorhabditis Elegans Propagation and Maintenance

The *C. elegans* strain N_2_ Bristol (wild type) was purchased from the *Caenorhabditis* Genetic Center (CGC), University of Michigan, USA. The nematodes were propagated on NGM agar plates, supplemented with *E. coli* OP50 as carbon source, and incubated at 20 °C [32].

### 2.3. Cultivation and Extracts Production

*S. aquimarina* was cultivated in all the media for the extracts production listed above. Pre-inocula were prepared by cultivating *S. aquimarina* in 3 mL of each selected medium in sterile tubes for 24 h at 20 °C, in orbital shaking at 210 rpm. After the incubation, the pre-inocula were inoculated into 500 mL flasks containing 125 mL of the appropriate medium at an initial concentration of 0.01 optical density (OD) at 600 nm. After five days of incubation at 20 °C, the cultures were centrifuged at 6800× *g* at 4 °C for 40 min, then the obtained supernatants and pellets were processed separately. Supernatants were extracted twice with two volumes of EtOAc, while the pellets were extracted three times with 10 mL of MeOH. Successively, both organic phases were collected and evaporated with a rotary evaporator (BUCHI R-100, BÜCHI Labortechnik AG, Postfach, Switzerland) to obtain crude extracts. Finally, all the obtained extracts were dissolved in DMSO at 50 mg/mL and stored at 4 °C to be tested for bioactivities.

### 2.4. Extract Fractionation

The extract obtained from a 2 L culture of strain *S. aquimarina* in liquid TYP was resuspended in the minimum possible amount of MeOH and subjected to fractionation using C18 solid phase extraction (SPE) cartridges (Macherey-Nagel, Duren, Germany), utilizing H_2_O, MeOH, and different mixtures of MeOH and H_2_O as eluents. Fractions eluted at 65% (F1), 90% (F2), and 100% MeOH (F3) were collected, dried, dissolved in DMSO at 20 mg/mL, and tested for biological assays.

### 2.5. Liquid Chromatography–High-Resolution Tandem Mass Spectrometry (LC-HRMS^2^)

The F2 fraction was resuspended in methanol (1 mg/mL) and analyzed through Liquid Chromatography–High-Resolution Tandem Mass Spectrometry (LC HRMS/MS). Experiments were performed using a Thermo LTQ Orbitrap XL high-resolution ESI mass spectrometer coupled to a Thermo U3000 HPLC system (Thermo Fisher Scientific, Waltham, MA, USA) and equipped with a 5-µm C18 column (50 × 2.10 mm, Kinetex) (Phenomenex, Torrance, CA, USA), as already described [33]. The column was eluted at 200 µL × min^−1^ with a gradient of 0.1% HCOOH supplemented with H_2_O (solvent A) and CH_3_CN (solvent B). The gradient program was set as follows: 3 min 5% A, 30 min from 5 to 99% B, and 7 min 100% B. A full MS scan event was acquired in positive ion mode. MS parameters were set as follows: spray voltage at 4.8 kV, capillary temperature at 285 °C, sheath gas rate at 32 units of N_2_ (ca. 150 mL × min^−1^), and the auxiliary gas rate at 15 units of N_2_ (ca. 50 mL/min). High-resolution tandem mass spectrometry (HRMS/MS) data were acquired in data-dependent acquisition mode. CID fragmentation was employed to obtain HRMS/MS scans, setting an isolation width of 2.0, normalized collision energy of 35, activation Q of 0.250, and an activation time of 30 ms.

### 2.6. Feature-Based Molecular Networking and Spectral Library Search

A molecular network was created with the Feature-Based Molecular Networking (FBMN) workflow [34] on GNPS [35], as previously reported [36]. MS data from the F2 fraction and the blank sample (MeOH) were first processed with MZMINE2 [37]. Briefly, mass detection was performed on raw data and centroided masses with mass level 1 and mass level 2, by keeping the noise level at 10,000 and 1000, respectively. The ADAP chromatogram algorithm was used to build chromatograms setting a minimum height of 1000 and *m*/*z* tolerance of 0.01 (or 20 ppm). As regards chromatogram deconvolution, the baseline cut-off algorithm was employed with the following settings: minimum height peak = 10,000, peak duration range = 0.0–6.0 min, baseline level = 1000, *m*/*z* range for MS2 scan = 0.05, and retention time range = 0.5 min. Chromatogram peaks were aligned by using the Join aligner algorithm (*m*/*z* tolerance at 0.001 or 5 ppm, absolute RT tolerance at 0.1 min). [M+Na–H], [M+K–H], [M+Mg−2H], [M+NH3], and [M+1, 13C] adducts were filtered out by setting the maximum relative height at 100%. In addition, peaks without associated MS2 spectra and peaks from the blank sample were filtered out from the peak list. Processed MS data were exported to GNPS for FBMN analysis. The molecular network was visualized using Cytoscape software version 3.7.2 [38]. The molecular networking job can be publicly accessed at https://gnps.ucsd.edu/ProteoSAFe/status.jsp?task=404e877578474ca6a43371e5d1410564.

### 2.7. Oil-Spreading Assay

The assay was conducted with some modifications from Morikawa et al. [39]. Briefly, 25 mL of water were settled in a Petri dish of 9 cm diameter and a thin oil layer was created by pouring 50 µL of exhaust motor oil on the water surface. Then, 1 µL of F2 at 10 mg/mL was dispensed onto the center of the oil surface. The presence of biosurfactants was revealed by the development of a clear zone in the oil layer. An amount of 1 µL of DMSO was used as a negative control.

### 2.8. Antibacterial Assay

Each pathogen was plated on MH agar (MHA) and incubated for 18 h at 37 °C. Then, a fresh colony was picked, inoculated in 3 mL of MH, and incubated for 18 h at 37 °C. Bacteria were then incubated in a fresh medium until log-phase (6 × 10^8^ CFU/mL) was reached. Serial dilutions were performed to obtain a final bacterial concentration of 1 × 10^5^ CFU/mL. The assay was conducted by broth microdilution assay. Briefly, 8 µL of each extract was added into 200 µL of MH at the initial concentration of 1000 µg/mL and serially 2-fold diluted. DMSO (2% *v*/*v*) represented the solvent control and is reported as vehicle on graphs. Plates were incubated for 18 h at 37 °C, and growth was measured with a Tecan plate reader (Tecan, Männedorf, Switzerland) by monitoring the OD_600_. The extract concentration that inhibited 95% of microbial growth was recorded as the minimum inhibitory concentration (MIC). The percentage of growth inhibition was obtained by the following formula:

(1) $$\%\;\mathrm{Growth}\;\mathrm{inhibition}=100\times \left[1\;-\;\frac{{\mathrm{OD}}_{600}\;\mathrm{of}\;\mathrm{the}\;\mathrm{treated}\;\mathrm{sample}}{{\mathrm{OD}}_{600}\;\mathrm{of}\;\mathrm{the}\;\mathrm{untreated}\;\mathrm{sample}}\right].$$

#### 2.8.1. MBC Assay

The minimal bactericidal concentration (MBC) was recorded as the lowest extract concentration killing 99.9% of the bacterial load after 24 h of incubation.

Briefly, after the determination of MIC, an aliquot of 50 µL was taken from ½ × MIC, 1 × MIC, and 2 × MIC wells and spread on MHA plates. Then, the plates were incubated for 24 h at 37 °C. After the incubation, the number of colony-forming units (CFUs) in the treated samples was compared with untreated samples. The lowest concentration that reduced CFU by 3-logarithmic growth (99.9%) was considered the MBC.

#### 2.8.2. Antimicrobial Synergy and Checkerboard Testing

Preliminary synergy screening of the extract was performed by modifying the Kirby–Bauer Disk Diffusion assay [40]. Briefly, the compound at MIC concentration was inoculated in 15 mL MHA and allowed to solidify in a Petri dish. Then, a fresh colony of MRSA was incubated on MH until 0.5 McFarland turbidity was reached. The bacterium was streaked on MHA, and 9 different antibiotic disks were placed on the agar plate. The plate was incubated at 37 °C, and the diameters of the inhibition areas were measured 18 h post incubation.

The combined effect of fosfomycin and extract was evaluated via checkerboard assays. Two-fold serial dilutions of the antibiotic and extract were assembled for the concentration ranges of 200–3.13 µg/mL and 200–0.39 µg/mL, respectively. A 2 × 10^5^ CFU/mL bacterial inoculum of MRSA was added to the single and combined compounds. The resulting plate was incubated at 37 °C for 24 h. The combinatorial inhibitory potential was listed as the fractional inhibitory concentration index (FICI), and calculated as follows:

(2) $$\mathrm{FICI}=\frac{\left(\mathrm{MIC}\;\mathrm{fosfomycin}\;\mathrm{in}\;\mathrm{combination}\right)}{\mathrm{MIC}\;\mathrm{fosfomycin}}+\frac{\left(\mathrm{MIC}\;F2\;\mathrm{in}\;\mathrm{combination}\right)}{\mathrm{MIC}\;F2}$$

Based on the obtained FICI, four scenarios were determined as follows:

- FICI ≤ 0.5, synergy;
- 0.5 < FICI ≤ 1.0, partial synergy;
- 1.0 < FICI ≤ 4.0, no interaction;
- FICI > 4.0, antagonism.

### 2.9. Antibiofilm Activity

As the formation of the biofilm proceeds in different steps, the antibiofilm activity towards *S. aureus* 6538 and MRSA was assessed with three experiments as follows:

#### 2.9.1. Biofilm Initial Cell Attachment Assay

After 18 h of cultivation, MRSA and *S. aureus* 6538 OD_600_ were adjusted to 0.1 (~8 × 10^7^ CFU/mL) in MHG and 200 µL dispensed in microtiter plates, and incubated for 1 h at 37 °C. After discarding the culture medium, non-adherent cells were removed by washing the plates twice with DPBS. MTT (20 µL) and MH (180 µL) were poured into each well of the plates, and incubated at 37 °C for 2 h [41]. Untreated bacteria (i.e., bacteria inoculated solely in MHG medium) were utilized as control. OD_450_ was determined, and attachment inhibition was calculated as follows:

(3) $$\%\;\mathrm{Attachment}\;\mathrm{inhibition}=100\times \left[1\;-\;\frac{{\mathrm{OD}}_{450}\;\mathrm{of}\;\mathrm{the}\;\mathrm{treated}\;\mathrm{sample}}{{\mathrm{OD}}_{450}\;\mathrm{of}\;\mathrm{the}\;\mathrm{untreated}\;\mathrm{sample}}\right]$$

#### 2.9.2. Biofilm Inhibition Assay

*S. aureus* culture was grown for 18 h and then was adjusted to 0.1 OD_600_. An amount of 200 µL of bacterial suspension was placed in each well of a 96-well microtiter plate. The fraction F2 was added to the wells at different concentrations, and the plate was incubated for 24 h at 37 °C. Then, after discarding the medium and washing the plate twice, the formed biofilm was stained with 0.1% (*w*/*v*) Crystal Violet (CV) and the plate was incubated at RT for 30 min. Then, the plates were rinsed twice with water to eliminate CV excess, and ethanol (95%) was added to solubilize CV embedded into the biofilm, incubating for 30 min at RT. Finally, CV absorbance was measured at 570 nm and the reduction in biofilm formation was obtained as follows:

(4) $$\%\;\mathrm{Biofilm}\;\mathrm{inhibition}=100\times \left[1\;-\;\frac{{\mathrm{OD}}_{570}\;\mathrm{of}\;\mathrm{the}\;\mathrm{treated}\;\mathrm{sample}}{{\mathrm{OD}}_{570}\;\mathrm{of}\;\mathrm{the}\;\mathrm{untreated}\;\mathrm{sample}}\right]$$

#### 2.9.3. Biofilm Degradation Assay

The biofilm degradation activity was evaluated by CV staining protocol [42]. *S. aureus* was inoculated in MHG for 24 h at 37 °C. The following day, the inoculum was adjusted to 10^8^ CFU/mL, and 100 µL was dispensed to each well of a 96-well plate. The biofilm formation was obtained by incubating the plate at 37 °C for 24 h. The wells filled with 100 µL of sterile medium represented the negative controls. After the incubation, the wells were washed twice with DPBS to remove non-adhesive planktonic bacteria. An amount of 100 µL of the active fraction was added at different concentrations in each well, and the plate was incubated at 37 °C for 24 h. After the incubation, the plate was washed twice with DPBS and stained with 0.1% CV for 40 min at RT. Then, it was washed three times with DPBS to remove excess dye. Adhered CV was solubilized with 95% ethanol for 20 min under orbital shaking at RT. Biofilm biomass was recorded by measuring absorbance at 570 nm using a TECAN microplate reader (Tecan, Männedorf, Switzerland), and the percentage of degradation was obtained as follows:

(5) $$\%\;\mathrm{Biofilm}\;\mathrm{degradation}=100\times \left[1\;-\;\frac{{\mathrm{OD}}_{570}\;\mathrm{of}\;\mathrm{the}\;\mathrm{treated}\;\mathrm{sample}}{{\mathrm{OD}}_{570}\;\mathrm{of}\;\mathrm{the}\;\mathrm{untreated}\;\mathrm{sample}}\right]$$

### 2.10. Antiviral Assays

Before testing the antiviral activity, the cytotoxicity of fraction F2 on Vero cells was assessed. The cells were seeded in microtiter plates at a 2 × 10^4^ cell/well density and incubated in a humidified atmosphere for 24 h at 37 °C. Then, cells were treated with F2 at concentrations ranging from 25 to 400 µg/mL and incubated for another 24 h. The cytotoxicity of the extracts was determined by MTT assay (Sigma-Aldrich, St. Louis, MO, USA). Briefly, cells were incubated for 3 h with MTT (0.5 mg/mL), then formazan crystals were dissolved with DMSO (100 µL). Untreated cells were used as negative controls. The viability of the cells was evaluated by recording the absorbance (Abs) at the wavelength of 570 nm with a TECAN M-200 reader (Tecan, Männedorf, Switzerland). The percentage of cell viability was calculated according to the following formula:

(6) $$\mathrm{Cell}\;\mathrm{viability}\;\left(\%\right)=\frac{\mathrm{Abs}\;\mathrm{sample}}{\mathrm{Abs}\;\mathrm{control}}\times 100$$

F2 antiviral activity was assessed by the plaque reduction assay [43]. Four tests were performed in the presence of the extract at concentrations ranging from 200 to 25 µg/mL in FBS-free medium. In all the following treatments, Vero cells were plated in 24-well plates at an initial density of 1.2 × 10^4^ cells/well and incubated for 24 h at 37 °C. Depending on the specific test, viruses were added at a multiplicity of infection (MOI) of 0.01 or 0.1 plaque-forming unit (PFU)/mL and incubated for 1 h at 37 °C. Pleconaril 10 µM was used as a positive control against PV-1. For the other viruses, an internal control, represented by M15RL rhamnolipids at 25 µg/mL [43], was utilized as a positive control in co-treatment and virus pre-treatment assays, while dextran sulfate (1 µM) and aciclovir (5 µM) were used for cell pre-treatment and post-treatment, respectively. The untreated infected cells represented the negative control. The antiviral activity was calculated as the percentage of plaque reduction in the treated sample compared to the untreated, as follows:

(7) $$\mathrm{Antiviral}\;\mathrm{Activity}=\left(1-\frac{\mathrm{number}\;\mathrm{of}\;\mathrm{plaques}\;\mathrm{in}\;\mathrm{treated}\;\mathrm{sample}}{\mathrm{number}\;\mathrm{of}\;\mathrm{plaques}\;\mathrm{in}\;\mathrm{untreated}}\right)\times 100$$

#### 2.10.1. Co-Treatment Assay

The cell monolayer was simultaneously treated with the extract and the virus at MOI of 0.01 PFU/mL for an incubation time of 1 h at 37 °C. Subsequently, the supernatant was removed, and the cell monolayer was washed with DPBS and incubated in a complete culture medium supplemented with 5% carboxymethylcellulose (CMC) (Sigma-Aldrich, St. Louis, MO, USA) for 48 h at 37 °C, in 5% CO_2_. After the incubation, the cells were washed twice with DPBS, fixed with 4% formaldehyde, and stained with 0.5% CV. Finally, the number of plaques was counted.

#### 2.10.2. Virus Pre-Treatment Assay

The virus (10^4^ PFU/mL) was incubated in FBS-free DMEM in the presence of different extract concentrations for 1 h at 37 °C. After the incubation, each batch was diluted 1:10 in a fresh medium to obtain a concentration of 10^3^ PFU/mL. The obtained mixtures were used to infect Vero cells monolayers. After 1 h, the monolayers were washed with DPBS and incubated for 48 h in a complete medium supplemented with 5% CMC. After the incubation, the cells were fixed and stained, and the percentage of inhibition was calculated as described above.

#### 2.10.3. Post-Treatment Assay

After infecting Vero cells (MOI 0.01) for 1 h at 37 °C, the excess virus particles were poured off by rinsing the plate with DPBS. Then, the cells were exposed to the F2 fraction for 1 h at 37 °C. Finally, after washing with DPBS, cells were incubated in DMEM medium for 48 h.

#### 2.10.4. Cell Pre-Treatment

The Vero cells monolayer was pre-treated for 1 h with the extract. Subsequently, the compound was removed, and each virus was added to a MOI of 0.1 pfu/mL for 1 h at 37 °C. After viral adsorption, the supernatant was removed, the cell monolayer was washed with DPBS, and incubated for 48 h in a fresh medium. Finally, the plate was processed and analyzed as in Formula (7).

### 2.11. Anthelmintic Activity

The effect of *S. aquimarina* active fraction on nematodes was evaluated on *C. elegans*. The assay was conducted in 96-well plates. Each well contained a 150 µL solution of M9 buffer, 5 µg/mL Cholesterol, and *E. coli* OP50 at the concentration of 0.5 OD/mL as carbon source. The fraction was added to each well at different concentrations. DMSO 1% (*v*/*v*) was used as control to evaluate the eventual solvent effect on nematodes and reported as vehicle in graphs. *C. elegans* was synchronized by bleaching treatment [32], and 20–40 L4 worms were transferred to each well and incubated at 20 °C for up to 48 h. The wells were scored for living worms every 24 h. A worm was considered dead when it no longer responded to touch. For statistical purposes, 3 replicates per trial were carried out with a unique egg preparation.

### 2.12. Statistical Analysis

All the experiments were carried out in triplicate. GraphPad Prism 9 for Windows ver. 9.0.0 (GraphPad Software, San Diego, CA, USA, www.graphpad.com) was utilized for the generation of the graphs and the statistical analysis of normalized data by using one-way and two-way ANOVA followed by Dunnett’s multiple comparisons. Differences were considered statistically significant if *p*-values < 0.05. The normality of the distributions has been checked using the Shapiro test and the homogeneity of variance has been checked with the Brown–Forsythe test.

## 3. Results and Discussion

### 3.1. Production and Extraction of Secondary Metabolites from S. aquimarina

*S. aquimarina* was cultured in eight different liquid media following an OSMAC approach to induce the production of the maximum number of natural products. The culture media were chosen to explore the effects of different carbon and nitrogen sources on the biosynthesis of secondary metabolites in rich and limiting nutrient conditions. The crude extracellular extracts were tested for antibacterial activity by liquid inhibition assays against bacterial pathogens. Only TYP extract showed antibacterial activity against Gram-positive bacteria, recording a MIC value of 250 µg/mL against *S. aureus* 6538 and *S. epidermidis*.

Few studies have focused on the antimicrobial activity of metabolic products of *Shewanella* spp. Gharaei et al. reported a considerable antibacterial and antibiofilm potential of *Shewanella algae* glycolipid biosurfactant against several pathogenic bacteria, recording MIC values of 12.5 mg/mL and biofilm degradation rates greater than 53% against *Bacillus cereus*, *Streptococcus pneumonia*, *Pseudomonas aeruginosa*, *Escherichia coli*, *Klebsiella pneumoniae*, and *Acinetobacter* sp. [44]. Likewise, Gong et al. showed a total growth inhibition when *Aspergillus* member pathogens were treated with 200 µg/mL of dimethyl trisulfide and 2,4-bis (1,1-dimethylethyl)-phenol, secondary metabolites produced by *Shewanella algae* [30]. Marine secondary metabolites could represent an untapped reservoir for the search for new compounds for the treatment of infectious diseases [14]. Moore et al. demonstrated that the strain *Shewanella halifaxensis* IRL548 produced the compound 2-benzyl-4-chlorophenol with antibacterial properties against *E. coli* and *V. haveyii* with IC_50_ of 54.9 µM and 41.2 µM, respectively [45].

### 3.2. Extract Fractionation and Structural Prediction of Alkaloids from the F2 Fraction

The TYP crude extract was fractionated by solid-phase extraction (SPE) with a C18 cartridge, and the obtained fractions were tested for antimicrobial activity against *S. aureus* 6538. The fractions F1 and F3 eluted at 65% and 100% MeOH, respectively, did not show antimicrobial activity at the tested concentrations. In contrast, the fraction F2 (90% MeOH) showed an inhibitory effect greater than the crude extract, with a MIC value of 100 µg/mL.

The chemical composition of the most bioactive fraction was assessed by a combined LC–HRMS/MS and molecular networking approach to reveal putative metabolites exerting antimicrobial effects. For untargeted chemical profiling, high-resolution tandem mass spectra were acquired in data-dependent scan mode (DDA) to fragment the five most intense ions of a full-scan mass spectrum. Tandem mass data were analyzed using the feature-based molecular networking (FBMN) tool, available on the GNPS online platform, to generate a molecular network and visually display the chemical space present in the bioactive fraction. Moreover, MS/MS spectra were submitted for a spectral library search against the GNPS database.

**Figure 1:**
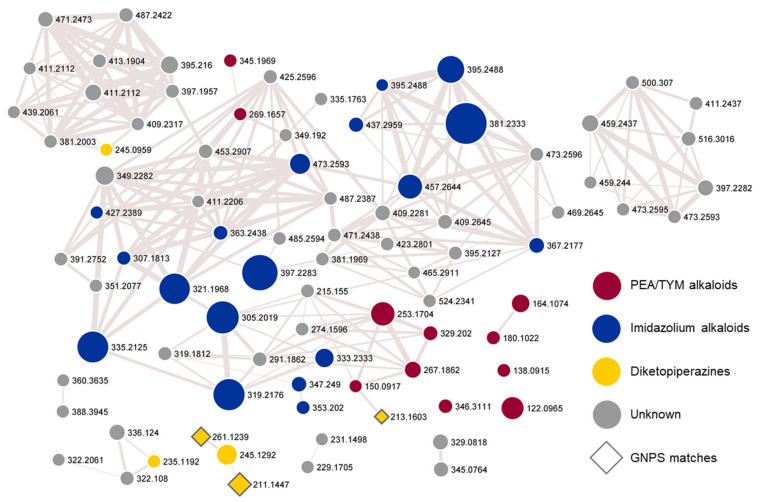
Molecular network of the 90% MeOH fraction from the TYP extracellular extract of *Shewanella aquimarina*. Nodes annotated in the present study are colored according to the relevant alkaloid class and their size is related to the metabolite amount. Edge thickness refers to the cosine score, which is indicative of the structural similarity of clustered metabolites. GNPS matches are highlighted with diamonds. PEA, phenethylamine; TYM, tyramine; GNPS, Global Natural Products Social Molecular Networking.

The molecular network (Figure 1) showed a large cluster of structurally related compounds. Manual inspection of the HRMS/MS fragmentation patterns of these metabolites disclosed the cluster to be mainly composed of phenethylamine (PEA)- and tyramine (TYM)-derived alkaloids (blue and red nodes). Most of them share neutral losses of C_8_H_8_ (104.0620 amu) and/or C_8_H_8_O (120.0570 amu), putatively corresponding to styrene and 4-hydroxystyrene, respectively, and indicative of PEA- and TYM-based derivatives (Figure S1). In addition, six diketopiperazines (DKPs) were found to be present in the network (orange nodes) (Table S1). The GNPS spectral library search allowed for the annotation of only three nodes (highlighted with diamonds in the network), belonging to the DKP group.

Overall, the structures of 42 alkaloids were predicted, thus leading to the identification of 29 putative new PEA- and TYM-derived alkaloids.

**Figure 2:**
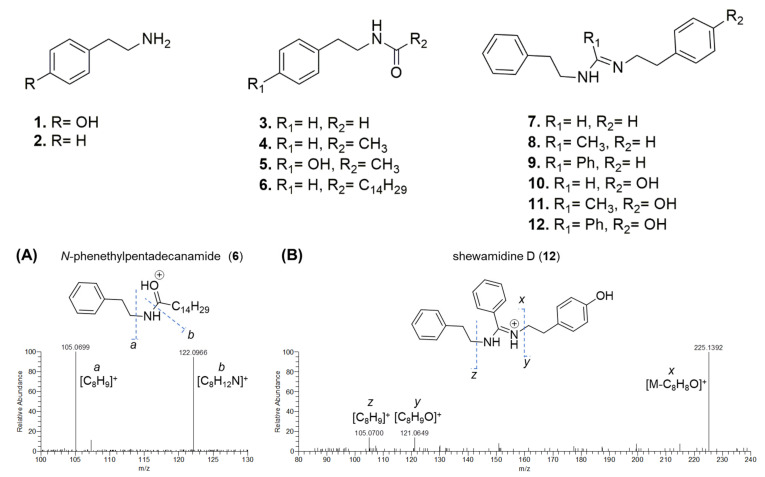
Structures of *N*-acyl phenethylamine and phenethylimidamide alkaloids from *S. aquimarina* (upper panel). Lower panel, HR-MS/MS spectra of the [M+H]^+^ pseudomolecular ions of (**A**) a representative *N*-acyl phenethylamine derivative, i.e., *N*-phenethylpentadecanamide (**6**), and (**B**) a representative imidamide alkaloid, i.e., shewamidine D (**12**) from *S. aquimarina*.

Dereplication of the bioactive fraction revealed the presence of four *N*-acyl phenethylamine derivatives (**3**–**6**, Table 2), together with their amine precursors, i.e., phenethylamine (**1**) and tyramine (**2**) (Figure 2). Mass tandem spectra of the [M+H]^+^ pseudomolecular ions of compounds **3**–**6** featured two abundant fragment ions, including (a) the [C_8_H_9_]^+^ ion deriving from the amide loss after *N*-Cα bond cleavage and (b) the 2-phenylethylammonium arising from the ketene loss (Figure 2A).

Moreover, the fraction contained six imidamide derivatives (**7**–**12**), five of them being new compounds (**8**–**12**) and tentatively named as shewamidines A-E (Table 2). Compound **7** was identified as *N*,*N*’-diphenethylformimidamide, previously isolated from the leaves of *Elaeocarpus tectorius* and reported as tectoramidine B [46]. Molecular formulas as well as fragmentation patterns of **7**–**12** were consistent with the structures of imidamide-based compounds (Figure 2B). HR MS^2^ spectra were dominated by the fragment ion *x*, generated by neutral loss of styrene (C_8_H_8_) or 4-hydroxystyrene (C_8_H_8_O), which, together with the fragment ions [C_8_H_9_]^+^ and/or [C_8_H_9_O]^+^, were indicative of the PEA- and/or TYM-derived moieties, respectively. Particularly, the fragment ion [C_8_H_9_O]^+^ generated after N−Cα bond cleavage in TYM by ESI in-source CID, has been reported to correspond to 6-hydroxyspiro[2.5]octadienylium [47]. The imidamide function and its R_1_ substituent in compounds **7**–**12** could be indirectly inferred from neutral losses leading to the formation of *z* and *y* fragments. As compared to tectoramidine B (**7**), shewamidines differ for the R_1_ and R_2_ substituent groups.

A comprehensive analysis of the HRMS/MS spectra of the [M+H]^+^ ions corresponding to blue nodes in the molecular network (Figure 1) revealed that the F2 fraction also contained putative novel PEA and TYM alkaloids bearing an imidazolium ring, which were tentatively named as shewazoles (**13**–**37**, Table 3). Indeed, these compounds share almost the same fragmentation pathways with discolins F-H from the marine flavobacterium *Tenacibaculum discolor* sv11 [48].

**Figure 3:**
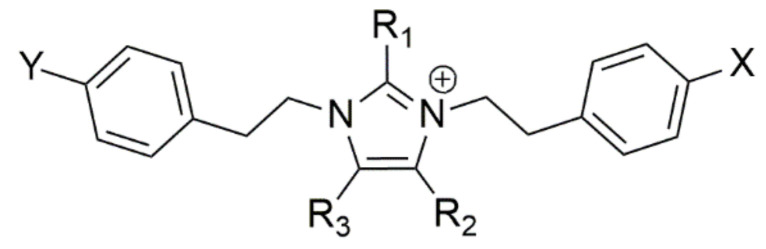
General structure of phenethylamine and tyramine alkaloids containing an imidazolium ring from *Shewanella aquimarina*.

The chemical structures of shewazoles were predicted to be composed of two PEA/TYM moieties, condensed together to form a central imidazolium ring, bearing alkyl and/or phenyl substituents (Figure 3).

**Figure 4:**
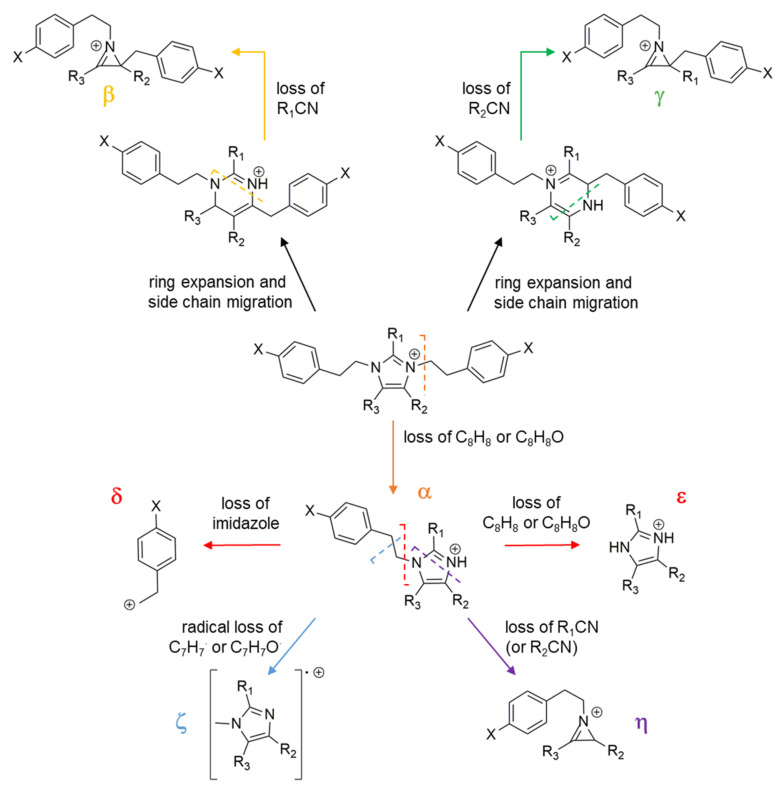
Proposed fragmentation pathway of phenethylamine and tyramine alkaloids containing an imidazolium ring from *S. aquimarina*.

**Figure 5:**
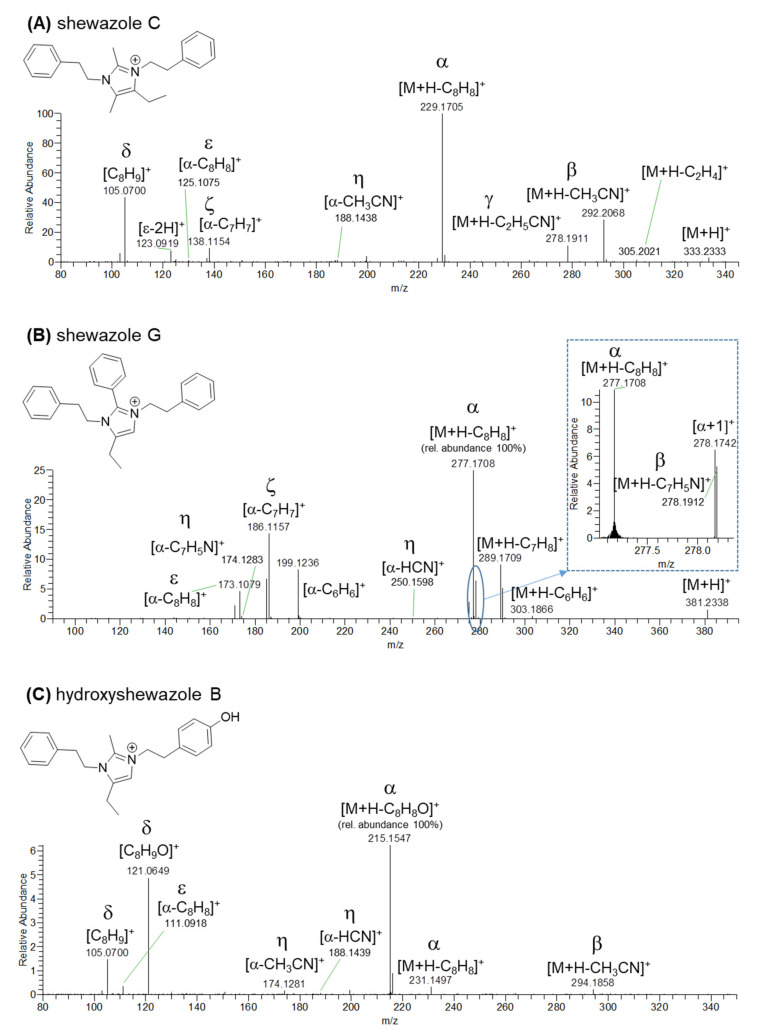
HR-MS/MS spectra of the [M+H]^+^ pseudomolecular ions of representative PEA/TYM alkaloids containing an imidazolium ring from *S. aquimarina*, i.e., (**A**) shewazole C (**17**), (**B**) shewazole G (**23**), and (**C**) hydroxyshewazole B (**31**). Fragment ions are labelled according to the proposed fragmentation pathway described in Figure 4.

The product ion spectra generated from the [M+H]^+^ ions of shewazoles are dominated by the fragment arising from neutral loss of C_8_H_8_ (104.0620 amu) or C_8_H_8_O (120.0570 amu) diagnostic of PEA or TYM (Figure 4 and Figure 5). Particularly, shewazoles having at least one TYM-derived motif display the [M+H-C_8_H_8_O]^+^ ion as the most abundant fragment (Figure 5C). The α fragmentation shown in Figure 4 is usually found in *N*-alkyl imidazoles and implies cleavage of the N-C bond with simultaneous proton transfer from the leaving styrene/hydroxystyrene moiety to the nitrogen [49]. In addition, the formation of the cognate δ ions, i.e., [C_8_H_9_]^+^ and/or [C_8_H_9_O]^+^, occurs during fragmentation of shewazoles, thus confirming the presence of PEA and/or TYM, as already observed for shewamidines (Figure 4 and Figure 5). Sequential losses of two styrene/hydroxystyrene molecules (C_8_H_8_/C_8_H_8_O) from the [M+H]^+^ ions generate the ε fragment ion corresponding to the central imidazolium core (Figure 4, fragmentation α + ε), as shown for discolins [48]. The presence of the imidazolium ring is also suggested by the homolytic scission reaction leading to formation of the radical cation ζ from the fragment ion α (Figure 4 and Figure 5).

An in-depth investigation of the MS tandem spectra allowed us to predict the substitution patterns of the central imidazolium scaffold as detailed in Table 3. As observed in discolins and substituted imidazoles [50], [M+H]^+^ pseudomolecular ions of shewazoles showed RCN and/or HCN losses which were useful to establish the alkyl and phenyl substituents directly linked to the imidazolium ring (Figure 4, fragmentations β and γ). A putative pathway leading to fragments arising from RCN and/or HCN losses may involve the formation of an ion species stabilized by ring expansion with concomitant side chain migration. These fragmentation pathways were supposed to produce azirine cations as described in Figure 4 [51]. Notably, neutral loss of R_1_CN generates a more abundant ion as compared to R_2_CN and R_3_CN losses according to MS/MS spectra recorded for discolins [48]. Therefore, this observation was used to assign the position of the R_1_ substituent group. Moreover, the α-type fragment ions of shewazoles were shown to undergo RCN loss through a similar mechanism, thereby yielding the η-type azirine cations, which allowed for the identification and position assignment of the R_1_, R_2_, and R_3_ groups (Figure 4 and Figure 5).

Additional low-abundance ions were useful for structural characterization of shewazoles. Particularly, [M+H]^+^ ions of shewazoles featuring phenyl substituents as well as the corresponding α fragments exhibited neutral loss of benzene (C_6_H_6_, 78.0470 amu) (Figure 5B). In addition, shewazoles often showed fragment ions arising from losses of the alkyl groups (i.e., ethyl, propyl, butyl) of the imidazolium ring as alkenes during ESI fragmentation (Figure 5A). HR-MS/MS spectra of the [M+H]^+^ pseudomolecular ions of representative PEA and TYM alkaloids containing an imidazolium ring from *S. aquimarina* are reported in Figure 5.

### 3.3. F2 Biosurfactant Activity

**Figure 6:**
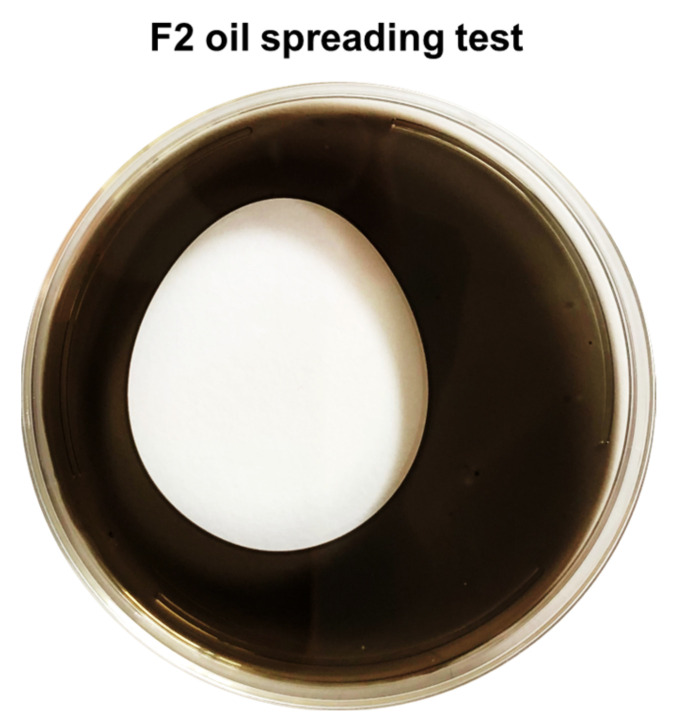
Oil spreading assay. The test relies on the biosurfactant’s ability to displace oil and form a clear zone in the oil layer.

Imidazolium alkaloids are widely reported as being surface active agents [52,53,54]. Therefore, the biosurfactant activity of the F2 fraction was subjected to the oil-spreading assay. This test reveals the presence of biosurfactants highlighted by a clear zone in the oil layer (Figure 6), comparable with other strong microbial biosurfactants [55].

### 3.4. F2 Antimicrobial Activity against Clinically Isolated S. aureus Strains

The bioactive potential of the F2 fraction was more deeply investigated. Since the TYP extract was active against *S. aureus*, we evaluated the antimicrobial activity against five clinical strains of *S. aureus*, which present several factors of virulence and resistance to antibiotics [56]. Again, F2 efficiently inhibited the growth of all the tested strains at 100 µg/mL (Table 4). Subsequently, the wells corresponding to the MIC, ½ × MIC, and 2 × MIC were used to check whether the fraction inhibited the growth of or killed the bacteria tested. Results showed that F2 is bactericidal at the MIC concentration against *S. aureus* 6538 and MRSA, killing the others at 2 × MIC. These findings are in accordance with the literature, in which imidazole alkaloids, such as discolins and bacillimidazoles, exhibited antimicrobial activity towards Gram-positive bacteria. Moreover, these antibacterial effects could be related to a biosurfactant activity, resulting in membrane disruption through a detergent-like mechanism of action [41,55].

**Figure 7:**
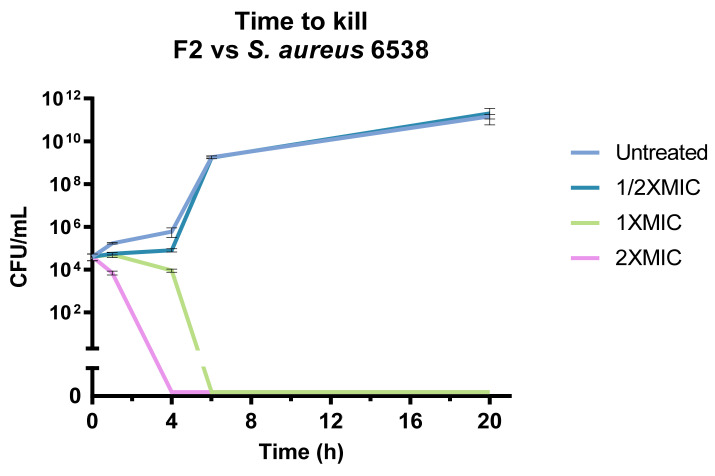
Time to kill of fraction F2 against *S. aureus* 6538. The fraction completely inhibited bacterial growth in 4 h at the MIC.

The antimicrobial activity of fraction F2 was further investigated on *S. aureus* ATCC 6538 in a time-course experiment (Figure 7). The experiment confirmed that the fraction is able to kill *S. aureus* within 6 h at the MIC and in 4 h at 2 × MIC, while we observe no bactericidal activity at ½ × MIC.

### 3.5. Synergistic Effect of Antibiotics and F2 on MRSA

**Figure 8:**
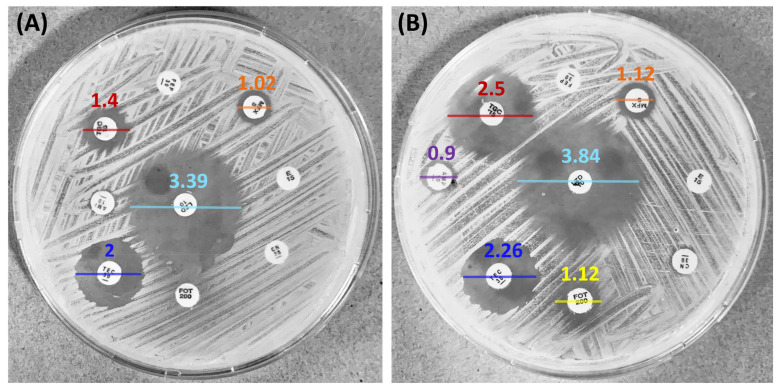
Kirby–Bauer synergy assay against MRSA, in which the effects of the antibiotics are represented by clear halos. In (**A**), the agar does not contain the active fraction, while in (**B**) the fraction F2 is present. The presence of the fraction increased the antibacterial activity of TGC (red), LZD (light blue), MFX (orange), AMP (purple), and TBG (dark blue). Notably, it completely restored the antibacterial activity of the fosfomycin (yellow).

Drug combinations can generate a synergistic effect aimed at avoiding the resistance of some multi-resistant pathogens and lowering the concentrations of single drugs [57]. The Kirby–Bauer test showed the synergistic effect given by F2 combined with different antibiotics. As a result, the inhibition halos of tigecycline (in red) increased from 1.4 to 2.5 cm and from 3.39 to 3.84 cm for linezolid (in light blue) (Figure 8).

**Figure 9:**
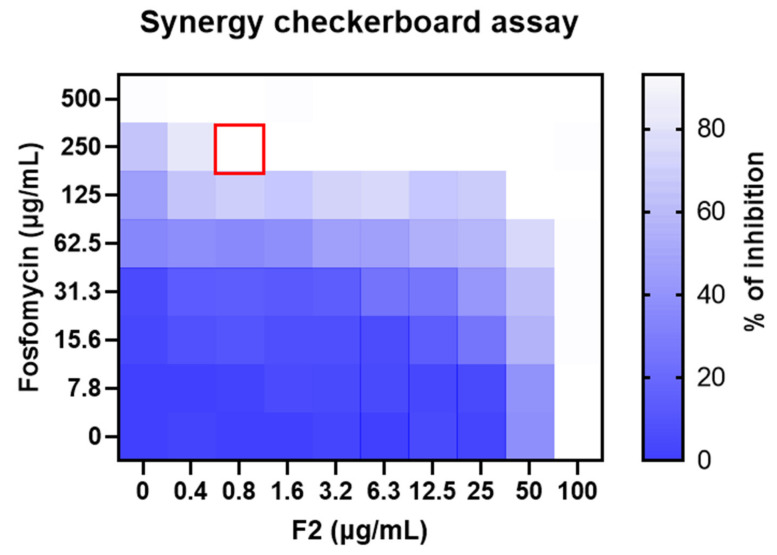
Checkerboard assay showing the combined effect of fosfomycin and the fraction F2 on MRSA. Columns contain 2-fold serial dilution of the fraction F2, while in the rows 2-fold dilutions of fosfomycin are presented. The heatmap is expressed as a percentage of bacterial growth reduction. The red square represents the combination with the lowest FICI.

Noteworthy is the restoration of MRSA sensitivity to fosfomycin (Figure 8, in yellow). Fosfomycin is a phosphonic acid derivative identified for the first time in 1969 [58]. Recently, fosfomycin is being favorably prescribed to treat severe infections also outside the urinary tract, its primary body district, even in combination with other drugs [59]. For this reason, we further investigated the combinatory effects through the checkerboard assay. The test revealed that the MIC value of fosfomycin against the MRSA strain is 500 µg/mL (Figure 9), while it is reduced to 250 µg/mL when combined with 0.8 µg/mL of F2. According to Equation (2), FICI resulted in 0.51, corresponding to a partial synergy.

As suggested previously, this result could also be due to the biosurfactant activity of F2. In fact, similar outcomes were obtained when fosfomycin was combined with daptomycin, a lipopeptide biosurfactant present on the market as antibiotic [60,61,62]. Some authors suggest that, by targeting the peptidoglycan, fosfomycin may increase the activity on the cell membrane [60,63]. In addition, marine-derived imidazole alkaloids have been already reported as antibiotic adjuvants, suppressing β-lactams and vancomycin resistance in resistant strains of *S. aureus* [64].

### 3.6. Antibiofilm Activity of F2 on S. aureus

Biofilm consists of bacterial communities enclosed within a polysaccharide matrix that guarantees resistance and protection against external agents; thus they are difficult to eradicate [65]. Biofilms are of great medical concern because they are resistant to antibiotic therapy, leading to persistent infections [66]. This is the case with infections caused by *S. aureus,* which is one of the leading pathogens in chronically infected wounds. Therefore, the action of the F2 fraction was evaluated on various phases of biofilm formation, such as initial attachment, maturation, and eradication of mature biofilm [67].

**Figure 10:**
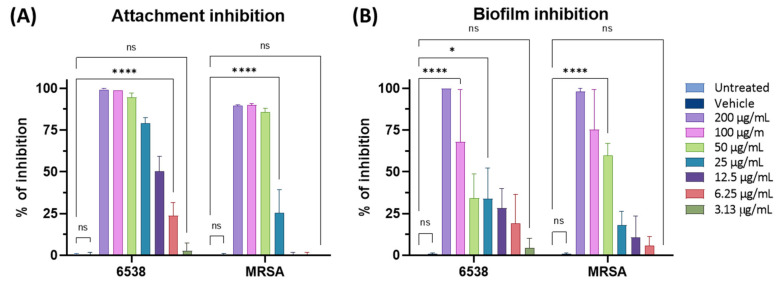
Antibiofilm activity of F2 against *S. aureus* 6538 and MRSA in two different steps: (**A**) the initial attachment to the substrate and (**B**) inhibition of mature biofilm formation. Two-way ANOVA was utilized for statistical analyses. Dunnett’s test was utilized for multiple comparisons. **** *p* < 0.0001, * *p* < 0.0332, ns: not statistically significant.

The results highlighted that F2 can prevent the initial attachment of *S. aureus* cells in the presence of a high bacterial load, acting in the first phase of biofilm formation (Figure 10A). F2 completely inhibited the attachment of *S. aureus* 6538 at 50 µg/mL, reducing it to 50% at 12.5 µg/mL. Even against MRSA strains, F2 reduced 90% of attached bacteria at 50 µg/mL. F2 also interfered in the second step of the biofilm life cycle. In fact, F2 completely inhibited the formation of the biofilm at 200 µg/mL and reduced it by 75% at 100 µg/mL against both 6538 and MRSA strains (Figure 10B). Notably, F2 is more effective on the biofilm formation of MRSA, recording 60% of biofilm inhibition at 50 µg/mL. This is a remarkable result, as the MRSA strain is a leading agent of nosocomial infections and biofilm is an additional virulence factor [56]. Moreover, even if some marine-derived pyrrole-imidazole alkaloids have been reported for antibiofilm activity, here we demonstrate for the first time the antibiofilm activity of phenethylamine and tyramine alkaloids [64]. Finally, the fraction is not able to eradicate a preformed mature biofilm (Figure S2).

### 3.7. Antiviral Activity of F2

A substantial part of emerging and re-emerging diseases in humans are of viral origin and caused mainly by enveloped viruses [68]. Here, F2’s antiviral potential has been explored against two herpes simplex viruses: HSV-1, which causes chronic infections mainly in the oral cavity and the face area, and HSV-2, which manifests mainly in the genital area [69]. The antiviral activity of the extract was first evaluated against HSV-1 with four treatments: virus pre-treatment (the viral particles were first treated with the fraction and then added to the cells), cell pre-treatment (the cells were first treated with the fraction and then infected), co-treatment (fraction and virus were added to the cell monolayer at the same time), and post-infection (the cells were infected, incubated, and then treated with the fraction).

**Figure 11:**
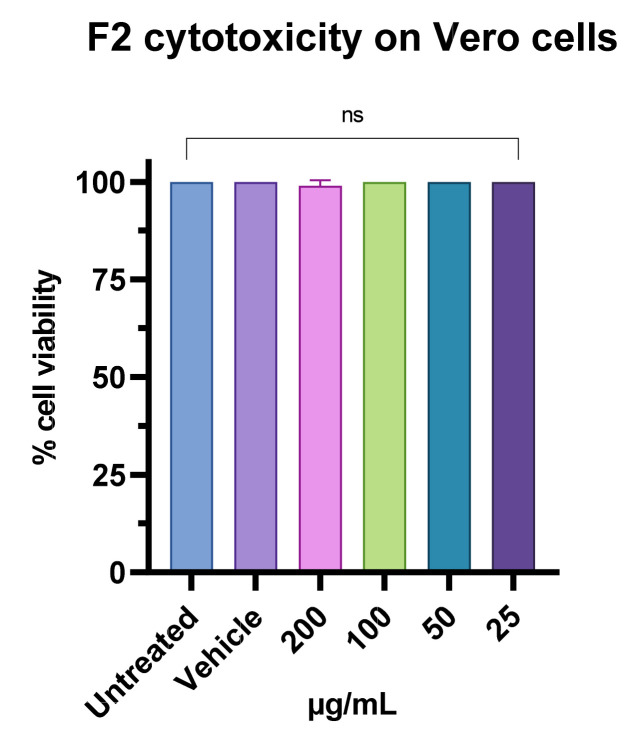
The toxicity of the F2 fraction was assessed through MTT on the Vero cells and expressed as a percentage of cell viability. One-way ANOVA was utilized for statistical analyses. Dunnett’s test was utilized for multiple comparisons. ns: not statistically significant.

Before testing the antiviral activity, the cytotoxicity of fraction F2 was evaluated through MTT assay on Vero cells. The cell monolayer was exposed to F2 at concentrations ranging from 25 to 400 µg/mL (Figure 11). After 24 h of treatment substantial reduction in cell viability was observed following exposure with the fraction F2 at the highest concentrations. In particular, the F2 fraction showed 30% vitality at 400µg/mL, while no toxicity was estimated at lower concentrations.

**Figure 12:**
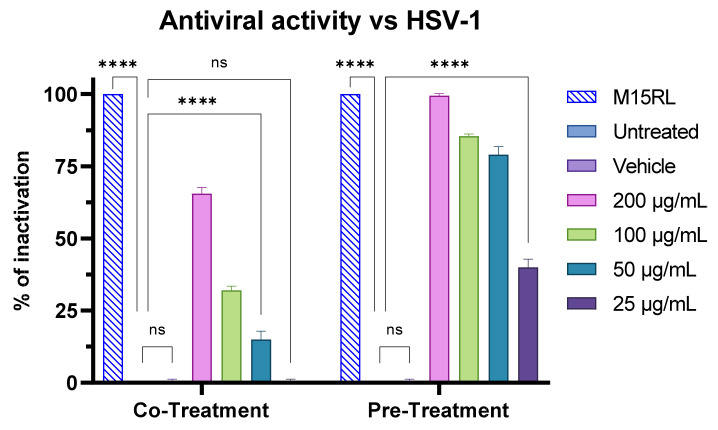
Antiviral activity of F2 against HSV-1 in the co-treatment and pre-treatment assays. M15RL at 25 µg/mL was utilized as positive control [43]. Two-way ANOVA was utilized for statistical analyses. Dunnett’s test was utilized for multiple comparisons. **** *p* < 0.0001, ns: not statistically significant.

Since the F2 fraction showed no toxicity below 200 µg/mL, its antiviral activity was investigated at concentrations ranging from 25 to 200 µg/mL. F2 exhibited a strong inhibitory effect in the virus pre-treatment assay, achieving an inhibition percentage ranging from 100% to 40% at the concentrations tested (Figure 12). In the co-treatment experiment, complete viral inactivation was not observed, recording only 70% of inactivation at 200 µg/mL. Furthermore, no activity was detected in post-infection and cell pre-treatment conditions (Figure S3).

**Figure 13:**
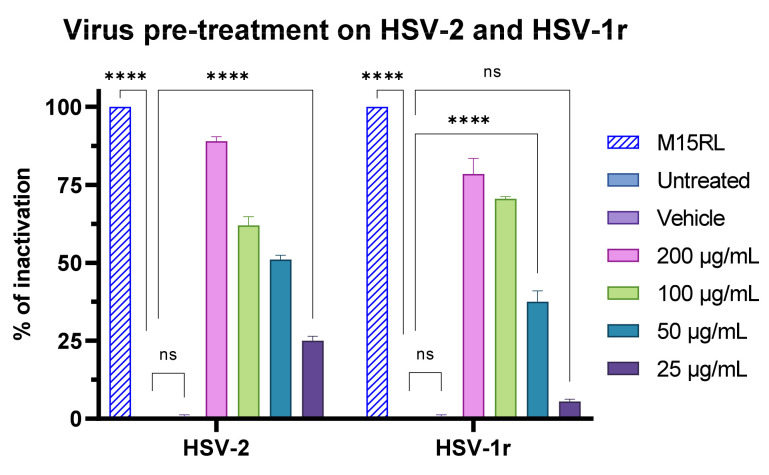
Antiviral activity of F2 against HSV-2 and HSV-1 resistant to acyclovir (HSV-1r) in the virus pre-treatment assay. M15RL at 25 µg/mL was utilized as positive control [43]. Two-way ANOVA was utilized for statistical analyses. Dunnett’s test was utilized for multiple comparisons. **** *p* < 0.0001; ns: not statistically significant.

After selection of the highest safe dose of F2, the virus pre-treatment assay was extended to other members of the *Herpesviridae* family, HSV-2 and HSV-1r. F2 showed lower activity against HSV-2 and HSV-1r than towards HSV-1, achieving 85% of viral inactivation on HSV-2 and 75% on HSV-1r at 200 µg/mL (Figure 13).

**Figure 14:**
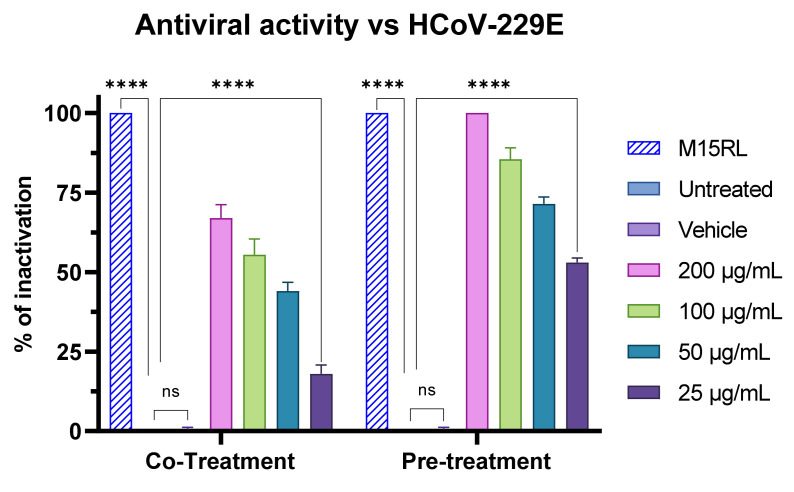
Antiviral activity of F2 against HcoV-229E in the co-treatment and pre-treatment assays. M15RL at 25 µg/mL was utilized as positive control [43]. Two-way ANOVA was utilized for statistical analyses. Dunnett’s test was utilized for multiple comparisons. **** *p* < 0.0001, ns: not statistically significant.

The highest antiviral activity achieved in the pre-treatment shows that F2 inactivated the virus before it could enter the host. These results suggest a mechanism of action targeting the viral membrane, which is consistent with the biosurfactant activity exhibited by the F2 fraction [43,70]. However, entry of herpesvirus into human cells is favored by specific glycoproteins that mediate the initial attachment and fusion of the membranes [71]. Therefore, to check whether F2 selectively targets some herpesvirus glycoproteins or acts nonspecifically on the viral membrane, we assessed its putative effects against a different enveloped virus, i.e., HCoV-229E. It belongs to the *Coronaviridae* family of ssRNA viruses, possessing a different repertoire of glycoproteins for the entry into the host as compared to herpesviruses [72]. Thus, the antiviral activity was investigated through all the previous four experiments described above. Once again, no inhibition was detected in cells pre-treatment and in post-infection assays (Figure S4), while in the virus pre-treatment and co-treatment assays antiviral activity was very similar to that observed against HSV-1 (Figure 14). Indeed, F2 showed 75% of viral inactivation in the co-treatment assay and complete inactivation in the virus pre-treatment assay at the highest concentration tested. The strong, non-selective inhibitory action in virus pre-treatment against both virus families indicates that the alkaloid mixture has a direct early effect on the envelope, resulting in the inability of the viral particles to merge with the cell membrane and to continue the stages of infection, and thus in their inactivation.

**Figure 15:**
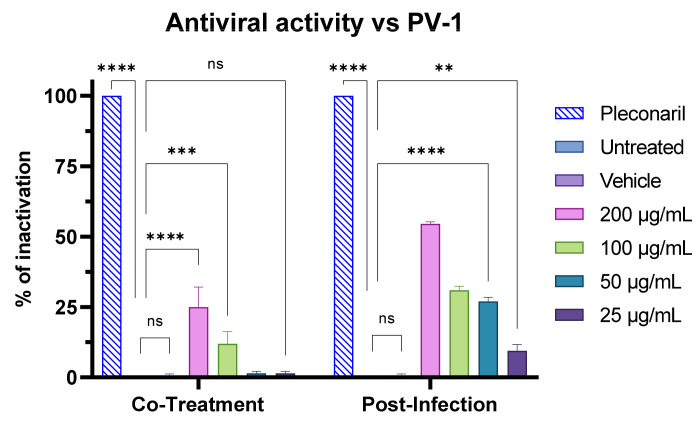
Antiviral activity of F2 against the poliovirus PV-1 in the co-treatment and post-treatment assays. Pleconaril 10 µM was used as positive control. Two-way ANOVA was utilized for statistical analyses. Dunnett’s test was utilized for multiple comparisons. **** *p* < 0.0001, *** *p* < 0.0002, ** *p* < 0.0021; ns: not statistically significant.

Finally, we performed further assays on a virus lacking an envelope. In particular, the activity of F2 was assessed against the poliovirus PV-1 through the four tests mentioned before. In accordance with biosurfactant activity focused against the viral membrane, results showed no activity in the virus pre-treatment (Figure S5). Interestingly, F2 showed 25% and 50% of viral inactivation at 200 µg/mL in the co-treatment and post-infection assays, respectively (Figure 15). This could be explained by a different mechanism of action exploited in the late phase of the viral life cycle.

However, little is known from the literature on the antiviral activities of these families of imidazolium alkaloids. Gong et al. reported weak antiviral activity against Influenza A virus H1N1 of an imidazolium alkaloid produced by the marine sponge *Pericharax heteroraphis* [73]. To the best of our knowledge, this is the first report of imidazolium alkaloids produced by a marine bacterium having activity against members of the *Herpesviridae*, *Coronaviridae*, and *Picornaviridae* families.

### 3.8. Anthelmintic Activity of F2 on C. elegans

**Figure 16:**
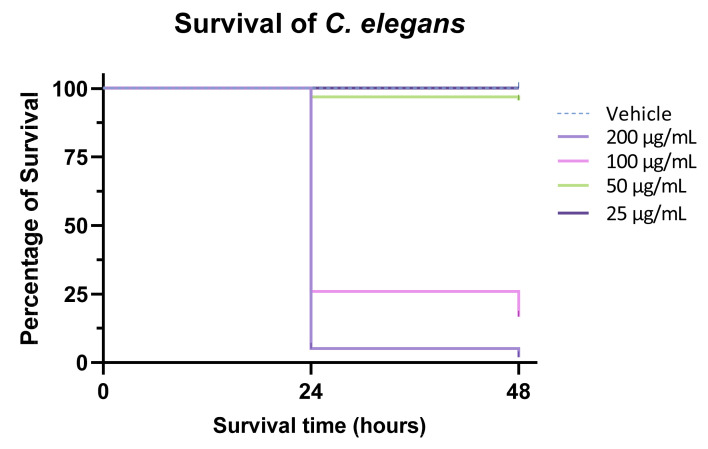
Survival of *C. elegans* after 24 h and 48 h of exposition to F2 fraction.

Alkaloids are known for their promising bioactivities towards parasites that cause the so-called “neglected tropical diseases” (NTDs) for which few actual drugs are available [74,75]. The anthelmintic effect of the alkaloids contained in the active fraction was evaluated in liquid survival assays against the nematode *C. elegans* as a model system [76]. We observed a significant effect of the active fraction on adult nematodes’ viability compared to controls (Figure 16). Specifically, at the concentration of 200 µg/mL the fraction showed almost 99% mortality of nematodes after 48 h, while all the worms remained viable in the negative control group (medium with 1% DMSO). At the concentration of 100 µg/mL we still observed significant activity with only 16% of the worms still viable after 48 h, while a lower concentration (50 µg/mL) showed poor activity (5% death rate). At the concentration of 25 µg/mL no effect on worms’ viability was detected.

Similar molecules have already shown anthelmintic activities. Rocha and colleagues have reported promising activities of imidazole alkaloids isolated from the leaves of the Brazilian plant *Pilocarpus microphyllus* [77]. One of these molecules, epiisopilosine, showed anthelmintic activity at a very low concentration (3.125 µg/mL) against *Schistosoma mansoni*, a plathelminth parasite. However, this is the first report of anthelmintic activities of imidazole alkaloids isolated from marine bacteria. Alkaloids are historically isolated from plants, but recently the number of bioactive alkaloids from marine sources has increased [78]. These results, together with ours, strongly indicate the potential of the oceans as a source of new molecules to cope with the emergency of NTDs.

## 4. Conclusions

In this study, we identified 29 novel phenethylamine- and tyramine-derived alkaloids through the LC-HRMS/MS molecular networking-based investigation of the most bioactive fraction from the marine bacterium *S. aquimarina*. Most of these molecules, namely shewazoles, have been demonstrated to be imidazolium alkaloids (24 out of 29 new compounds). Although some related analogues have been already described from *Tenacibaculum discolor* sv11 [25,48] and a sponge-associated *Bacillus* strain [26], shewazoles stand out as displaying a different substitution pattern on the imidazolium core. Indeed, several congeners bear a phenyl group on the central imidazolium ring, never reported before for this natural-product family.

The enriched fraction of these molecules showed antibacterial, antibiofilm, antiviral, and anthelmintic activities and enhanced the antimicrobial effects of antibiotics currently included in clinical settings, thus representing a valid ally in the fight against microbial drug resistance. Notably, we proved here for the first time that this alkaloid mixture inactivates members of the *Herpesviridae* and *Coronaviridae* families, likely disrupting the viral envelope as a cationic surfactant. Finally, as no specific anti-picornavirus drugs have yet been approved by the US Food and Drug Administration (FDA) [79], the observed anti-poliovirus effects in the late stages of the viral life cycle make these alkaloids promising lead compounds for treatment therapies, hence deserving further investigation. 

## Figures and Tables

**Table 1:** *S. aureus* isolates investigated for antimicrobial activity.

*Bacterial* sp.	Strain Code	Resistance Phenotype	Source
*S. aureus*	MSSA	Multisensitive	Eye
*S. aureus*	β-lactamase	Beta-lactamase producer	Wound
*S. aureus*	MLSB	Constitutive resistance to macrolides	Blood
*S. aureus*	QRSA	Quinolones resistance	Sputum
*S. aureus*	MRSA	Methicillin resistance	Blood

**Table 2:** *N*-acyl phenethylamine and phenethylimidamide alkaloids from *Shewanella aquimarina*.

Compound	*R*_t_	[M+H]^+^	*m*/*z*
tyramine (**1**)	1.1	C_8_H_12_NO	138.0915
phenylethylamine (**2**)	1.7	C_8_H_12_N	122.0965
*N*-(4-hydroxyphenethyl)acetamide (**5**)	5.7	C_10_H_14_NO_2_	180.1022
*N*-formyl-2-phenylethylamine (**3**)	11.1	C_9_H_12_NO	150.0917
*N*-acetyl-2-phenylethylamine (**4**)	11.6	C_10_H_14_NO	164.1071
shewamidine A (**10**)	12.2	C_17_H_21_N_2_O	269.1656
shewamidine B (**11**)	12.9	C_18_H_23_N_2_O	283.1813
*N*,*N*’-diphenethylformimidamide (**7**)	14.6	C_17_H_21_N_2_	253.1704
shewamidine C (**8**)	15.3	C_18_H_23_N_2_	267.1861
shewamidine D (**12**)	15.4	C_23_H_25_N_2_O	345.1969
shewamidine E (**9**)	17.6	C_23_H_25_N_2_	329.2019
*N*-phenethylpentadecanamide (**6**)	32.3	C_23_H_39_NO	346.3111

**Table 3:** PEA and TYM alkaloids containing an imidazolium ring from *Shewanella aquimarina*.

Imidazolium PEA Alkaloids								
Compound	*R*_t_ (min.)	[M+H]^+^	*m*/*z*	R_1_	R_2_	R_3_	X	Y
shewazole A (**13**) ^a^	15.8	C_20_H_23_N_2_	291.1862	H	CH_3_	H	H	H
bacillimidazole A (**14**) [26]	16.5	C_21_H_25_N_2_	305.2017	H	CH_3_	CH_3_	H	H
shewazole B (**15**)	17.1	C_22_H_27_N_2_	319.2174	CH_3_	H	C_2_H_5_	H	H
isoshewazole B (**16**) ^a^	17.5	C_22_H_27_N_2_	319.2174	H	C_2_H_5_	CH_3_	H	H
shewazole C (**17**)	17.9	C_23_H_29_N_2_	333.2333	CH_3_	C_2_H_5_	CH_3_	H	H
isoshewazole C (**18**) ^a^	18.5	C_23_H_29_N_2_	333.2333	C_3_H_7_	CH_3_	H	H	H
shewazole D (**19**)	18.5	C_25_H_25_N_2_	353.2018	H	Ph	H	H	H
shewazole E (**20**)	18.8	C_26_H_27_N_2_	367.2178	Ph	CH_3_	H	H	H
shewazole F (**21**)	18.9	C_24_H_31_N_2_	347.2490	CH_3_	C_3_H_7_	CH_3_	H	H
isoshewazole F (**22**) ^a^	19.3	C_24_H_31_N_2_	347.2490	C_4_H_9_	CH_3_	H	H	H
shewazole G (**23**)	19.4	C_27_H_29_N_2_	381.2331	Ph	H	C_2_H_5_	H	H
shewazole H (**24**) ^a^	19.5	C_25_H_33_N_2_	361.2645	C_4_H_9_	CH_3_	CH_3_	H	H
shewazole I (**25**)	19.8	C_28_H_31_N_2_	395.2488	Ph	C_2_H_5_	CH_3_	H	H
isoshewazole I (**26**)	20.0	C_28_H_31_N_2_	395.2488	Ph	C_3_H_7_	H	H	H
shewazole J (**27**)	21.6	C_33_H_33_N_2_	457.2643	Ph	Ph	C_2_H_5_	H	H
shewazole K (**28**)	21.8	C_31_H_37_N_2_	437.2958	C_4_H_9_	Ph	C_2_H_5_	H	H
**Imidazolium TYM Alkaloids**								
**Compound**	***R*_t_ (min.)**	**[M+H]^+^**	***m*/*z***	**R_1_**	**R_2_**	**R_3_**	**X**	**Y**
hydroxyshewazole A (**29**) ^b^	13.5	C_20_H_23_N_2_O	307.1809	H	CH_3_	H	OH ^c^	H
hydroxybacillimidazole A (**30**) ^b^	14.3	C_21_H_25_N_2_O	321.1967	H	CH_3_	CH_3_	OH	H
hydroxyshewazole B (**31**)	14.8	C_22_H_27_N_2_O	335.2122	CH_3_	H	C_2_H_5_	OH	H
dihydroxyshewazole I (**32**)	16.0	C_28_H_31_N_2_O_2_	427.2388	Ph	C_2_H_5_	CH_3_	OH	OH
hydroxyshewazole F (**33**)	16.8	C_24_H_31_N_2_O	363.2438	CH_3_	C_3_H_7_	CH_3_	OH ^c^	H
hydroxyshewazole H (**34**) ^a^	17.3	C_25_H_33_N_2_O	377.2596	C_4_H_9_	CH_3_	CH_3_	OH	H
hydroxyshewazole G (**35**) ^b^	17.4	C_27_H_29_N_2_O	397.2280	Ph	H	C_2_H_5_	OH ^c^	H
hydroxyshewazole I (**36**) ^a^	17.9	C_28_H_31_N_2_O	411.2440	Ph	C_2_H_5_	CH_3_	OH ^c^	H
hydroxyshewazole J (**37**) ^b^	19.7	C_33_H_33_N_2_O	473.2593	Ph	Ph	C_2_H_5_	OH ^c^	H

^a^ Not present in the molecular network. ^b^ Substitution patterns have been assigned according to the corresponding imidazolium PEA alkaloids. ^c^ The OH group could not be assigned unambiguously to position X or Y.

**Table 4:** Antimicrobial activity of F2 fraction vs. different *S. aureus* strains.

F2 Minimal Inhibitory and Bactericidal Concentrations (µg/mL)		
*S. aureus* Strain	MIC	MBC
ATCC 6538	100	100
MLSB	100	200
Quinolone res.	100	200
Β-lactamase	100	200
MSSA	100	200
MRSA	100	100

## Data Availability

Not applicable.

## Supplementary Materials

The following supporting information can be downloaded at: https://www.mdpi.com/article/10.3390/pharmaceutics15082139/s1, Table S1: Diketopiperazines from Shewanella aquimarina, Figure S1: Neutral loss/precursor *m*/*z* plot from HR-MS/MS data of the F2 fraction; Figure S2: Biofilm degradation; Figure S3: Antiviral activity vs. HSV-1; Figure S4: Antiviral activity vs. HCoV-229E; Figure S5: Antiviral activity vs. PV-1, Figure S6: MRSA antibiogram, Figure S7: MSSA antibiogram, Figure S8: β-Lactamase antibiogram, Figure S9: QRSA antibiogram, Figure S10: MLSB antibiogram.

## Abbreviations

AMP	Ampicillin
CFUs	Colonies forming units
CMC	Carboxymethylcellulose
CN	Gentamycin
CV	Crystal violet
DKPs	Diketopiperazines
DMEM	Dulbecco’s Modified Eagle Medium
DMSO	Dimethyl sulfoxide
DPBS	Dulbecco’s phosphate buffered saline
E	Erythromycin
EtOAc	Ethyl acetate
FBMN	Feature-Based Molecular Networking
FBS	Fetal Bovine Serum
FEP	Cefepime
FICI	Fractional inhibitory concentration index
FOT	Fosfomycin
HCoVs	Human coronaviruses
HRMS/MS	High-resolution tandem mass spectrometry
HSV-1	Herpes simplex virus type 1
HSV-1r	HSV-1 acyclovir-resistant
HSV-2	Herpes simplex virus type 2
IHME	Institute for Health Metrics and Evaluation
LC HRMS/MS	Liquid Chromatography–High-Resolution Tandem Mass Spectrometry
LZD	Linezolid
MB	Marine Broth
MBC	Minimal bactericidal concentration
MeOH	Methanol
MFX	Moxifloxacin
MH	Muller Hinton
MIC	Minimum inhibitory concentration
MLSB	Constitutive resistance to macrolides
MOI	Multiplicity of infection
MRSA	Methicillin resistance
MSM	Mineral Salt Medium
MSSA	Multisensitive
MTT	3-(4,5-dimethylthiazol-2-yl)-2,5-diphenyl tetrazolium bromide
NGM	Nematode Growth Medium
NTDs	Neglected tropical diseases
OD	Optical density
OSMAC	One strain many compounds
PEA	Phenethylamine
PFU	Plaque-forming unit
PV-1	Poliovirus type-1
QRSA	Quinolones resistance
RT	Room Temperature
SARS-CoV-2	Severe Acute Respiratory Syndrome coronavirus 2
SPE	Solid phase extraction
TEC	Teicloplanin
TGB	Thioglycollate
TGC	Tigecycline
TSB	Trypticasein Soy Broth
TYM	Tyramine

## References

[1] Roser M., Ritchie H., Spooner F. (2021). Burden of Disease. https://ourworldindata.org/burden-of-disease.

[2] Craft K.M., Nguyen J.M., Berg L.J., Townsend S.D. (2019). Methicillin-resistant *Staphylococcus aureus* (MRSA): Antibiotic-resistance and the biofilm phenotype. MedChemComm.

[3] Abraham L., Bamberger D.M. (2020). *Staphylococcus aureus* Bacteremia: Contemporary Management. Mo. Med..

[4] Kranjec C., Morales Angeles D., Torrissen Marli M., Fernandez L., Garcia P., Kjos M., Diep D.B. (2021). Staphylococcal Biofilms: Challenges and Novel Therapeutic Perspectives. Antibiotics.

[5] Burrell C.J., Howard C.R., Murphy F.A. (2017). Epidemiology of Viral Infections. Fenner and White’s Medical Virology.

[6] Malary M., Abedi G., Hamzehgardeshi Z., Afshari M., Moosazadeh M. (2016). The prevalence of herpes simplex virus type 1 and 2 infection in Iran: A meta-analysis. Int. J. Reprod. Biomed..

[7] Jiang Y.C., Feng H., Lin Y.C., Guo X.R. (2016). New strategies against drug resistance to herpes simplex virus. Int. J. Oral Sci..

[8] Liu D.X., Liang J.Q., Fung T.S. (2021). Human Coronavirus-229E, -OC43, -NL63, and -HKU1 (*Coronaviridae*). Encyclopedia of Virology.

[9] Karthikeyan A., Joseph A., Nair B.G. (2022). Promising bioactive compounds from the marine environment and their potential effects on various diseases. J. Genet. Eng. Biotechnol..

[10] Costello M.J., Chaudhary C. (2017). Marine Biodiversity, Biogeography, Deep-Sea Gradients, and Conservation. Curr. Biol..

[11] Rotter A., Gaudêncio S.P., Klun K., Macher J.-N., Thomas O.P., Deniz I., Edwards C., Grigalionyte-Bembič E., Ljubešić Z., Robbens J. (2021). A New Tool for Faster Construction of Marine Biotechnology Collaborative Networks. Front. Mar. Sci..

[12] Zhou Q., Hotta K., Deng Y., Yuan R., Quan S., Chen X. (2021). Advances in Biosynthesis of Natural Products from Marine Microorganisms. Microorganisms.

[13] König G.M., Kehraus S., Seibert S.F., Abdel-Lateff A., Müller D. (2006). Natural Products from Marine Organisms and Their Associated Microbes. ChemBioChem.

[14] Srinivasan R., Kannappan A., Shi C., Lin X. (2021). Marine Bacterial Secondary Metabolites: A Treasure House for Structurally Unique and Effective Antimicrobial Compounds. Mar. Drugs.

[15] De Santi C., Tedesco P., Ambrosino L., Altermark B., Willassen N.P., de Pascale D. (2014). A new alkaliphilic cold-active esterase from the psychrophilic marine bacterium *Rhodococcus* sp.: Functional and structural studies and biotechnological potential. Appl. Biochem. Biotechnol..

[16] Hurdle J.G., O’Neill A.J., Chopra I., Lee R.E. (2011). Targeting bacterial membrane function: An underexploited mechanism for treating persistent infections. Nat. Rev. Microbiol..

[17] Bjerk T.R., Severino P., Jain S., Marques C., Silva A.M., Pashirova T., Souto E.B. (2021). Biosurfactants: Properties and Applications in Drug Delivery, Biotechnology and Ecotoxicology. Bioengineering.

[18] Tedesco P., Maida I., Palma Esposito F., Tortorella E., Subko K., Ezeofor C.C., Zhang Y., Tabudravu J., Jaspars M., Fani R. (2016). Antimicrobial Activity of Monoramnholipids Produced by Bacterial Strains Isolated from the Ross Sea (Antarctica). Mar. Drugs.

[19] Gallimore W., Badal S., Delgoda R. (2017). Chapter 18—Marine Metabolites: Oceans of Opportunity. Pharmacognosy.

[20] Abbas S., Mahmoud H. (2022). Identification of Sponge-Associated Bacteria from the Coast of Kuwait and Their Potential Biotechnological Applications. Front. Microbiol..

[21] Qasim M.S., Lampi M., Heinonen M.K., Garrido-Zabala B., Bamford D.H., Kakela R., Roine E., Sarin L.P. (2021). Cold-Active *Shewanella glacialimarina* TZS-4(T) nov. Features a Temperature-Dependent Fatty Acid Profile and Putative Sialic Acid Metabolism. Front. Microbiol..

[22] Teli P., Sahiba N., Sethiya A., Soni J., Agarwal S. (2022). Imidazole derivatives: Impact and prospects in antiviral drug discovery. Imidazole-Based Drug Discovery.

[23] Cui B., Zheng B.L., He K., Zheng Q.Y. (2005). Imidazole Alkaloids from Lepidium meyenii and Methods of Usage.

[24] Mingzhang A., Wenwen J., Longjang Y. (2010). Application of Maca Imidazole Alkaloid in Preparation of Cardiovascular Drugs.

[25] Wang L., Linares-Otoya V., Liu Y., Mettal U., Marner M., Armas-Mantilla L., Willbold S., Kurtán T., Linares-Otoya L., Schäberle T.F. (2022). Discovery and Biosynthesis of Antimicrobial Phenethylamine Alkaloids from the Marine Flavobacterium *Tenacibaculum discolor* sv11. J. Nat. Prod..

[26] Yan J.-X., Wu Q., Helfrich E.J.N., Chevrette M.G., Braun D.R., Heyman H., Ananiev G.E., Rajski S.R., Currie C.R., Clardy J. (2022). Bacillimidazoles A-F, Imidazolium-Containing Compounds Isolated from a Marine Bacillus. Mar. Drugs.

[27] Jin Z. (2011). Muscarine, imidazole, oxazole, and thiazole alkaloids. Nat. Prod. Rep..

[28] Tanaka N., Kusama T., Takahashi-Nakaguchi A., Gonoi T., Fromont J., Kobayashi J. (2013). Nagelamides X–Z, Dimeric Bromopyrrole Alkaloids from a Marine Sponge *Agelas* sp.. Org. Lett..

[29] Zhang F., Wang B., Prasad P., Capon R.J., Jia Y. (2015). Asymmetric Total Synthesis of (+)-Dragmacidin D Reveals Unexpected Stereocomplexity. Org. Lett..

[30] Gong A.D., Li H.P., Shen L., Zhang J.B., Wu A.B., He W.J., Yuan Q.S., He J.D., Liao Y.C. (2015). The Shewanella algae strain YM8 produces volatiles with strong inhibition activity against *Aspergillus* pathogens and aflatoxins. Front. Microbiol..

[31] Franci G., Folliero V., Cammarota M., Zannella C., Sarno F., Schiraldi C., de Lera A.R., Altucci L., Galdiero M. (2018). Epigenetic modulator UVI5008 inhibits MRSA by interfering with bacterial gyrase. Sci. Rep..

[32] Stiernagle T. (2006). Maintenance of *C. elegans*. WormBook.

[33] Della Sala G., Coppola D., Virgili R., Vitale G.A., Tanduo V., Teta R., Crocetta F., de Pascale D. (2022). Untargeted Metabolomics Yields Insights into the Lipidome of *Botrylloides niger* Herdman, 1886, An Ascidian Invading the Mediterranean Sea. Front. Mar. Sci..

[34] Nothias L.-F., Petras D., Schmid R., Dührkop K., Rainer J., Sarvepalli A., Protsyuk I., Ernst M., Tsugawa H., Fleischauer M. (2020). Feature-based molecular networking in the GNPS analysis environment. Nat. Meth..

[35] Wang M., Carver J.J., Phelan V.V., Sanchez L.M., Garg N., Peng Y., Nguyen D.D., Watrous J., Kapono C.A., Luzzatto-Knaan T. (2016). Sharing and community curation of mass spectrometry data with Global Natural Products Social Molecular Networking. Nat. Biotechnol..

[36] Scarpato S., Teta R., Della Sala G., Pawlik J.R., Costantino V., Mangoni A. (2020). New Tricks with an Old Sponge: Feature-Based Molecular Networking Led to Fast Identification of New Stylissamide L from *Stylissa caribica*. Mar. Drugs.

[37] Pluskal T., Castillo S., Villar-Briones A., Orešič M. (2010). MZmine 2: Modular framework for processing, visualizing, and analyzing mass spectrometry-based molecular profile data. BMC Bioinform..

[38] Shannon P., Markiel A., Ozier O., Baliga N.S., Wang J.T., Ramage D., Amin N., Schwikowski B., Ideker T. (2003). Cytoscape: A software environment for integrated models of biomolecular interaction networks. Genome Res..

[39] Morikawa M., Hirata Y., Imanaka T. (2000). A study on the structure–function relationship of lipopeptide biosurfactants. Biochim. Biophys. Acta Mol. Cell. Biol. Lipids.

[40] CLSI (2018). Methods for Dilution Antimicrobial Susceptibility Tests for Bacteria that Grow Aerobically.

[41] Buonocore C., Giugliano R., Della Sala G., Palma Esposito F., Tedesco P., Folliero V., Galdiero M., Franci G., de Pascale D. (2023). Evaluation of Antimicrobial Properties and Potential Applications of *Pseudomonas gessardii* M15 Rhamnolipids towards Multiresistant *Staphylococcus aureus*. Pharmceutics.

[42] Folliero V., Franci G., Dell’Annunziata F., Giugliano R., Foglia F., Sperlongano R., De Filippis A., Finamore E., Galdiero M. (2021). Evaluation of Antibiotic Resistance and Biofilm Production among Clinical Strain Isolated from Medical Devices. Int. J. Microbiol..

[43] Giugliano R., Buonocore C., Zannella C., Chianese A., Palma Esposito F., Tedesco P., De Filippis A., Galdiero M., Franci G., de Pascale D. (2021). Antiviral Activity of the Rhamnolipids Mixture from the Antarctic Bacterium *Pseudomonas gessardii* M15 against Herpes Simplex Viruses and Coronaviruses. Pharmaceutics.

[44] Gharaei S., Ohadi M., Hassanshahian M., Porsheikhali S., Forootanfar H. (2022). Isolation, Optimization, and Structural Characterization of Glycolipid Biosurfactant Produced by Marine Isolate *Shewanella algae* B12 and Evaluation of Its Antimicrobial and Anti-biofilm Activity. Appl. Biochem. Biotechnol..

[45] Moore S.L., Berthomier L., Braganza C.D., MacKichan J.K., Ryan J.L., Visnovsky G., Keyzers R.A. (2016). Identification, library synthesis and anti-vibriosis activity of 2-benzyl-4-chlorophenol from cultures of the marine bacterium *Shewanella halifaxensis*. Bioorg. Med. Chem. Lett..

[46] Ezeoke M.C., Krishnan P., Sim D.S., Lim S.H., Low Y.Y., Chong K.W., Lim K.H. (2018). Unusual phenethylamine-containing alkaloids from *Elaeocarpus tectorius*. Phytochemistry.

[47] Asakawa D., Sugiyama E., Mizuno H., Todoroki K. (2021). Study of Substituted Phenethylamine Fragmentation Induced by Electrospray Ionization Mass Spectrometry and Its Application for Highly Sensitive Analysis of Neurotransmitters in Biological Samples. J. Am. Soc. Mass Spectrom..

[48] Wang L., Marner M., Mettal U., Liu Y., Schäberle T.F. (2022). Seven New Alkaloids Isolated from Marine Flavobacterium *Tenacibaculum discolor* sv11. Mar. Drugs.

[49] Lesimple A., Mamer O., Miao W., Chan T.H. (2006). Electrospray mass spectral fragmentation study of N,N′-disubstituted imidazolium ionic liquids. J. Am. Soc. Mass Spectrom..

[50] Cooper G., Irwin W.J. (1975). Mass spectra of some substituted imidazoles. Org. Mass Spectrom..

[51] Bowie J., Cooks R., Lawesson S., Schroll G. (1967). Electron impact studies. XII. Mass spectra of substituted imidazoles. Aust. J. Chem..

[52] Wang D., Galla H.J., Drucker P. (2018). Membrane interactions of ionic liquids and imidazolium salts. Biophys. Rev..

[53] Chiba R., Ito M., Nishio Y. (2010). Addition effects of imidazolium salts on mesophase structure and optical properties of concentrated hydroxypropyl cellulose aqueous solutions. Polymer. J..

[54] Modaressi A., Sifaoui H., Mielcarz M., Domańska U., Rogalski M. (2007). Influence of the molecular structure on the aggregation of imidazolium ionic liquids in aqueous solutions. Colloid Surf. Physicochem. Eng Asp..

[55] Buonocore C., Tedesco P., Vitale G.A., Palma Esposito F., Giugliano R., Monti M.C., D’Auria M.V., de Pascale D. (2020). Characterization of a New Mixture of Mono-Rhamnolipids Produced by *Pseudomonas gessardii* Isolated from Edmonson Point (Antarctica). Mar. Drugs.

[56] Tong S.Y., Davis J.S., Eichenberger E., Holland T.L., Fowler V.G. (2015). *Staphylococcus aureus* infections: Epidemiology, pathophysiology, clinical manifestations, and management. Clin. Microbiol. Rev..

[57] Cheng Y.-S., Williamson P.R., Zheng W. (2019). Improving therapy of severe infections through drug repurposing of synergistic combinations. Curr. Opin. Pharmacol..

[58] Hendlin D., Stapley E.O., Jackson M., Wallick H., Miller A.K., Wolf F.J., Miller T.W., Chaiet L., Kahan F.M., Foltz E.L. (1969). Phosphonomycin, a New Antibiotic Produced by Strains of *Streptomyces*. Science.

[59] Antonello R.M., Principe L., Maraolo A.E., Viaggi V., Pol R., Fabbiani M., Montagnani F., Lovecchio A., Luzzati R., Di Bella S. (2020). Fosfomycin as Partner Drug for Systemic Infection Management. A Systematic Review of Its Synergistic Properties from In Vitro and In Vivo Studies. Antibiotics.

[60] Lee Y.-C., Chen P.-Y., Wang J.-T., Chang S.-C. (2019). A study on combination of daptomycin with selected antimicrobial agents: In vitro synergistic effect of MIC value of 1 mg/L against MRSA strains. BMC Pharmacol. Toxicol..

[61] Lingscheid T., Poeppl W., Bernitzky D., Veletzky L., Kussmann M., Plasenzotti R., Burgmann H. (2015). Daptomycin plus fosfomycin, a synergistic combination in experimental implant-associated osteomyelitis due to methicillin-resistant *Staphylococcus aureus* in rats. Antimicrob. Agents Chemother..

[62] Kügler J.H., Le Roes-Hill M., Syldatk C., Hausmann R. (2015). Surfactants tailored by the class Actinobacteria. Front Microbiol..

[63] Dhand A., Bayer A.S., Pogliano J., Yang S.-J., Bolaris M., Nizet V., Wang G., Sakoulas G. (2011). Use of antistaphylococcal β-lactams to increase daptomycin activity in eradicating persistent bacteremia due to methicillin-resistant *Staphylococcus aureus*: Role of enhanced daptomycin binding. J. Clin. Infect. Dis..

[64] Melander R.J., Liu H.B., Stephens M.D., Bewley C.A., Melander C. (2016). Marine sponge alkaloids as a source of anti-bacterial adjuvants. Bioorg. Med. Chem. Lett..

[65] Crouzet M., Le Senechal C., Brözel V.S., Costaglioli P., Barthe C., Bonneu M., Garbay B., Vilain S. (2014). Exploring early steps in biofilm formation: Set-up of an experimental system for molecular studies. BMC Microbiol..

[66] Muhammad M.H., Idris A.L., Fan X., Guo Y., Yu Y., Jin X., Qiu J., Guan X., Huang T. (2020). Beyond Risk: Bacterial Biofilms and Their Regulating Approaches. Front. Microbiol..

[67] Verderosa A.D., Totsika M., Fairfull-Smith K.E. (2019). Bacterial Biofilm Eradication Agents: A Current Review. Front. Chem..

[68] Seidah N.G., Pasquato A., Andréo U. (2021). How Do Enveloped Viruses Exploit the Secretory Proprotein Convertases to Regulate Infectivity and Spread?. Viruses.

[69] Rathbun M.M., Szpara M.L. (2021). A holistic perspective on herpes simplex virus (HSV) ecology and evolution. Adv. Virus Res..

[70] Palma Esposito F., Giugliano R., Della Sala G., Vitale G.A., Buonocore C., Ausuri J., Galasso C., Coppola D., Franci G., Galdiero M. (2021). Combining OSMAC Approach and Untargeted Metabolomics for the Identification of New Glycolipids with Potent Antiviral Activity Produced by a Marine *Rhodococcus*. Int. J. Mol. Sci..

[71] Connolly S.A., Jardetzky T.S., Longnecker R. (2021). The structural basis of herpesvirus entry. Nat. Rev. Microbiol..

[72] Klasse P.J., Bron R., Marsh M. (1998). Mechanisms of enveloped virus entry into animal cells. Adv. Drug Deliv. Rev..

[73] Gong K.K., Tang X.L., Liu Y.S., Li P.L., Li G.Q. (2016). Imidazole Alkaloids from the South China Sea Sponge *Pericharax heteroraphis* and Their Cytotoxic and Antiviral Activities. Molecules.

[74] Wang G.X., Zhou Z., Jiang D.X., Han J., Wang J.F., Zhao L.W., Li J. (2010). In vivo anthelmintic activity of five alkaloids from *Macleaya microcarpa* (Maxim) Fedde against *Dactylogyrus intermedius* in *Carassius auratus*. Vet. Parasitol..

[75] Williams A.R., Ropiak H.M., Fryganas C., Desrues O., Mueller-Harvey I., Thamsborg S.M. (2014). Assessment of the anthelmintic activity of medicinal plant extracts and purified condensed tannins against free-living and parasitic stages of *Oesophagostomum dentatum*. Parasites Vectors.

[76] Hahnel S.R., Dilks C.M., Heisler I., Andersen E.C., Kulke D. (2020). *Caenorhabditis elegans* in anthelmintic research—Old model, new perspectives. Int. J. Parasitol. Drugs Drug Resist..

[77] Rocha J.A., Andrade I.M., Véras L.M., Quelemes P.V., Lima D.F., Soares M.J., Pinto P.L., Mayo S.J., Ivanova G., Rangel M. (2017). Anthelmintic, Antibacterial and Cytotoxicity Activity of Imidazole Alkaloids from *Pilocarpus microphyllus* Leaves. Phytother. Res..

[78] Tempone A.G., Pieper P., Borborema S.E.T., Thevenard F., Lago J.H.G., Croft S.L., Anderson E.A. (2021). Marine alkaloids as bioactive agents against protozoal neglected tropical diseases and malaria. Nat. Prod. Rep..

[79] Wolthers K.C., Susi P., Jochmans D., Koskinen J., Landt O., Sanchez N., Palm K., Neyts J., Butcher S.J. (2019). Progress in human picornavirus research: New findings from the AIROPico consortium. Antivir. Res..

