# Supplementary material for: New Imidazolium Alkaloids with Broad Spectrum of Action from the Marine Bacterium Shewanella aquimarina
Source: Pharmaceutics. 2023 Aug 14;15(8):2139. doi: 10.3390/pharmaceutics15082139 (PMC10458398; doi:10.3390/pharmaceutics15082139)
Supplement: Supplementary file 1 [file pharmaceutics-15-02139-s001.zip › pharmaceutics-2530693-supplementary.pdf]

pharmaceutics

Supplementary Materials

New Imidazolium Alkaloids with Broad Spectrum of Action from the Marine Bacterium *Shewanella aquimarina*

Rosa Giugliano [1,†], Gerardo Della Sala [2,†], Carmine Buonocore [2,†], Carla Zannella [1], Pietro Tedesco [2], Fortunato Palma Esposito [2], Costanza Ragozzino [2,3], Annalisa Chianese [1], Maria Vittoria Morone [1], Valerio Mazzella [4,5], Laura Núñez-Pons [5,6], Veronica Folliero [7], Gianluigi Franci [7], Anna De Filippis [1], Massimiliano Galdiero [1,*] and Donatella de Pascale [2,*]

1 Department of Experimental Medicine, University of Campania "Luigi Vanvitelli", 80138 Naples, Italy; rosa.giugliano@unicampania.it (R.G.); carla.zannella@unicampania.it (C.Z.); annalisa.chianese@unicampania.it (A.C.); mariavittoria.morone@unicampania.it (M.V.M.); anna.defilippis@unicampania.it (A.D.F.)
2 Department of Ecosustainable Marine Biotechnology, Stazione Zoologica Anton Dohrn, Via Ammiraglio Acton, 55, 80133 Naples, Italy; gerardo.dellasala@szn.it (G.D.S.); carmine.buonocore@szn.it (C.B.); pietro.tedesco@szn.it (P.T.); fortunato.palmaesposito@szn.it (F.P.E.); costanza.ragozzino@studenti.unime.it (C.R.)
3 Department of Chemical, Biological, Pharmaceutical and Environmental Sciences, University of Messina, Viale F. Stagno d'Alcontres 31, 98166 Messina, Italy
4 Department of Integrative Marine Ecology (EMI), Stazione Zoologica Anton Dohrn, Ischia Marine Centre, Ischia, 80077 Naples, Italy; valerio.mazzella@szn.it
5 NBFC, National Biodiversity Future Center, 90133 Palermo, Italy; laura.nunezpons@szn.it
6 Department of Integrative Marine Ecology (EMI), Stazione Zoologica Anton Dohrn, Villa Comunale, 80121 Napoli, Italy
7 Department of Medicine, Surgery and Dentistry "Scuola Medica Salernitana", University of Salerno, 84081 Baronissi, Italy; vfolliero@unisa.it (V.F.); gfranci@unisa.it (G.F.)
* Correspondence: massimiliano.galdiero@unicampania.it (M.G.); donatella.depascale@szn.it (D.d.P.)
† These authors contributed equally to this work.

Contents 2

Table S1: Diketopiperazines from *Shewanella aquimarina* 3
Figure S1. Neutral loss/precursor *m/z* plot from HR-MS/MS data of the F2 fraction 3
Figure S2. Biofilm degradation 4
Figure S3. Antiviral activity vs HSV-1 4
Figure S4. Antiviral activity vs HCoV-229E 5
Figure S5: Antiviral activity vs PV-1 5
Figure S6: MRSA antibiogram 6
Figure S7: MSSA antibiogram 7
Figure S8: β—Lactamase antibiogram 8
Figure S9: QRSA antibiogram 9
Figure S10: MLSB antibiogram 10

Table S1. Diketopiperazines from *Shewanella aquimarina*.

**Diketopiperazines from F2**			
Diketopiperazine	R_t	[M+H]+	m/z
cyclo(Leu-Val)	11.9	$C_{11}H_{19}N_2O_2$	213.1603
cyclo(Met-Pro), *S*-Oxide	1.4	$C_{10}H_{16}N_2SO_3$	245.0959
cyclo(Phe-Pro)	11.4	$C_{14}H_{17}N_2O_2$	245.1290
cyclo(Tyr-Pro)	4.9	$C_{14}H_{17}N_2O_3$	261.1239
cyclo(Ile-Pro)	9.4	$C_{11}H_{19}N_2O_2$	211.1447
cyclo(His-Pro)	0.8	$C_{11}H_{15}N_4O_2$	235.1192

Neutral loss/precursor *m/z* plot

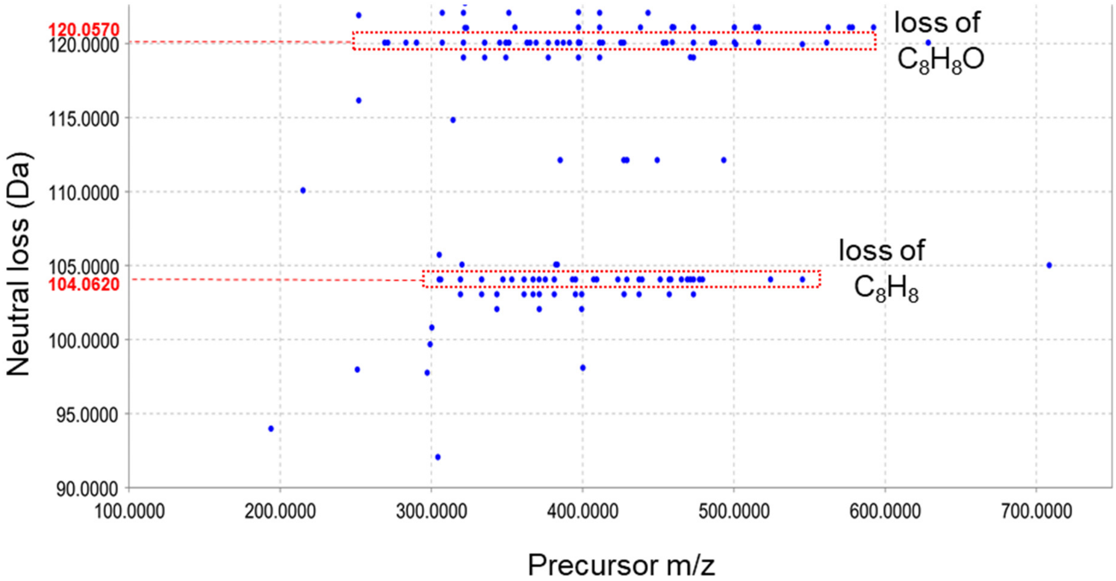

Figure S1. Neutral loss/precursor *m/z* plot from HR-MS/MS data of the F2 fraction from *Shewanella aquimarina*. Precursor ions sharing neutral losses of C_8H_8 (104.0620 Da) and/or C_8H_8O (120.0570 Da), are highlighted in red.

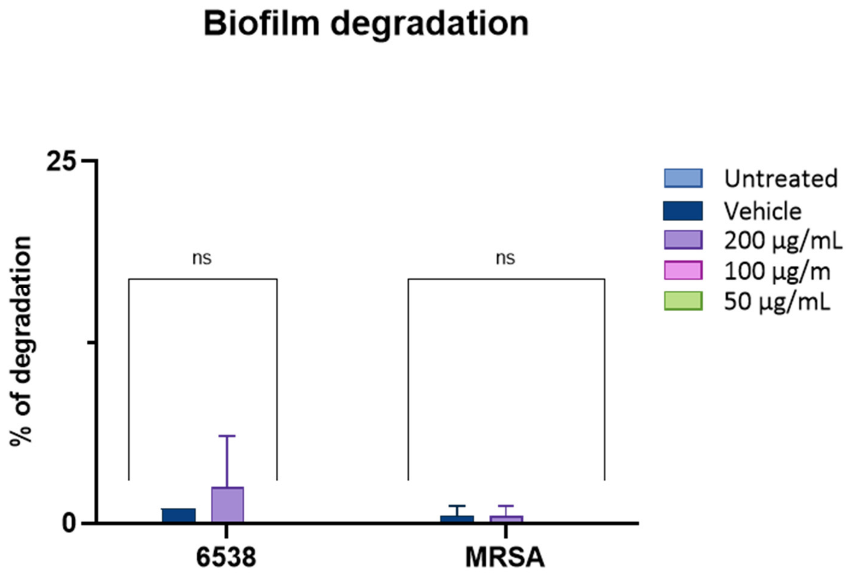

Figure S2. Biofilm degradation activity of F2 against *S. aureus* 6538 and MRSA. Statistical analyses were determined by two-way ANOVA with Dunnett's test for multiple comparisons referred to the untreated bacteria. ns (not statistically significant).

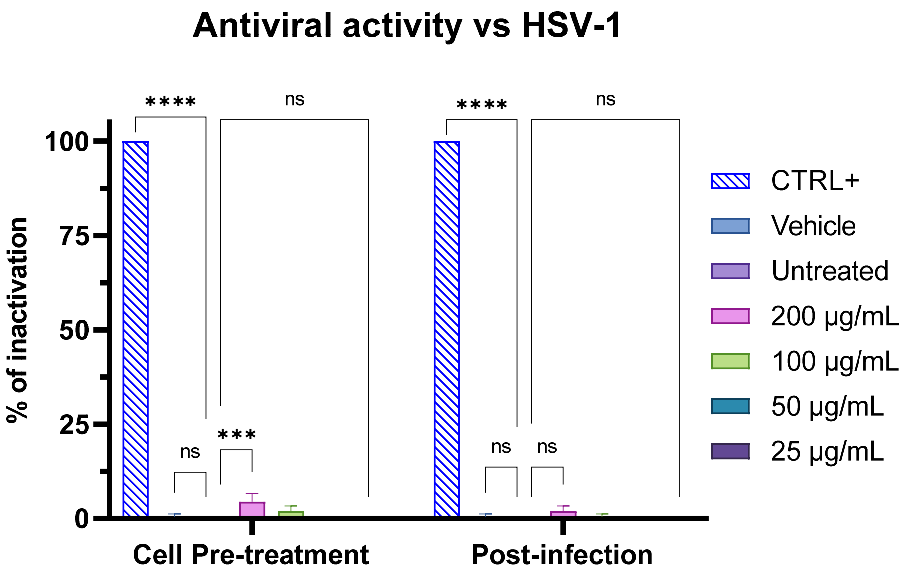

Figure S3. Antiviral activity of F2 against HSV-1 in the cell pre-treatment and post-infection assays. Dextran sulfate (1 µM) and aciclovir (5 µM) were used for cell pretreatment and post-treatment, respectivelly. Statistical analyses were determined by two-way ANOVA with Dunnett's test for multiple comparisons referred to the untreated infected cells **** $p < 0.0001$, *** $p < 0.0002$, ns (not statistically significant).

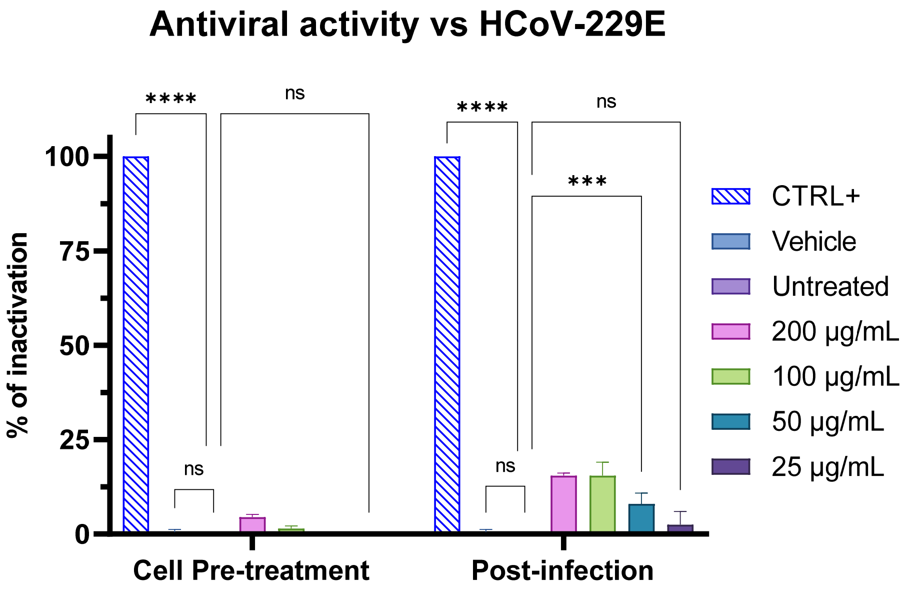

Figure S4. Antiviral activity of F2 against HCoV-229E in the cell pre-treatment and post-infection assays. Dextran sulfate (1 µM) and aciclovir (5 µM) were used for cell pretreatment and post-treatment, respectivelly. Statistical analyses were determined by two-way ANOVA with Dunnett's test for multiple comparisons referred to the untreated infected cells **** $p < 0.0001$, *** $p < 0.0002$, ns (not statistically significant).

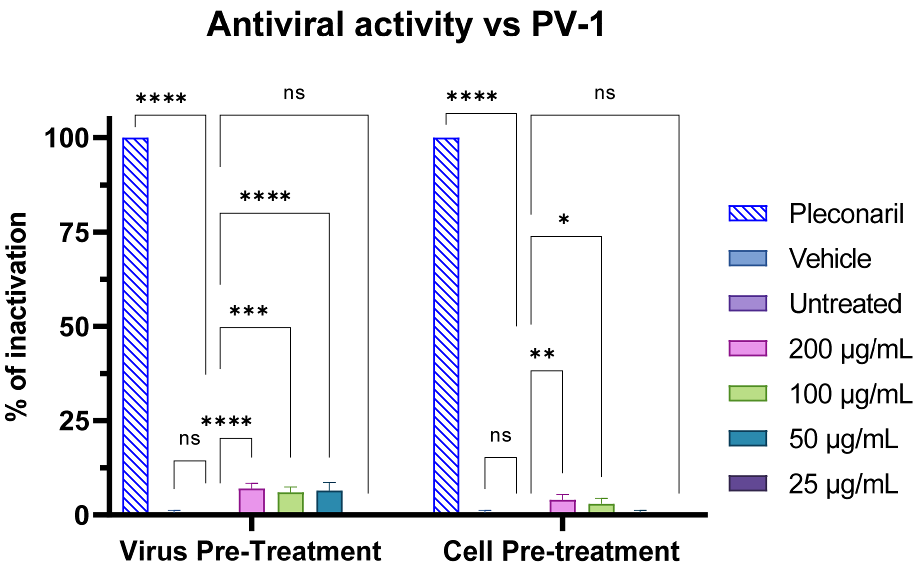

Figure S5. Antiviral activity of F2 against PV-1 in the virus and cell pre-treatment assays. Pleconaril 10 µM was used as positive control. Statistical analyses were determined by two-way ANOVA with Dunnett's test for multiple comparisons referred to the untreated infected cells **** $p < 0.0001$, *** $p < 0.0002$, ** $p < 0.0021$, * $p < 0.0332$, ns (not statistically significant).

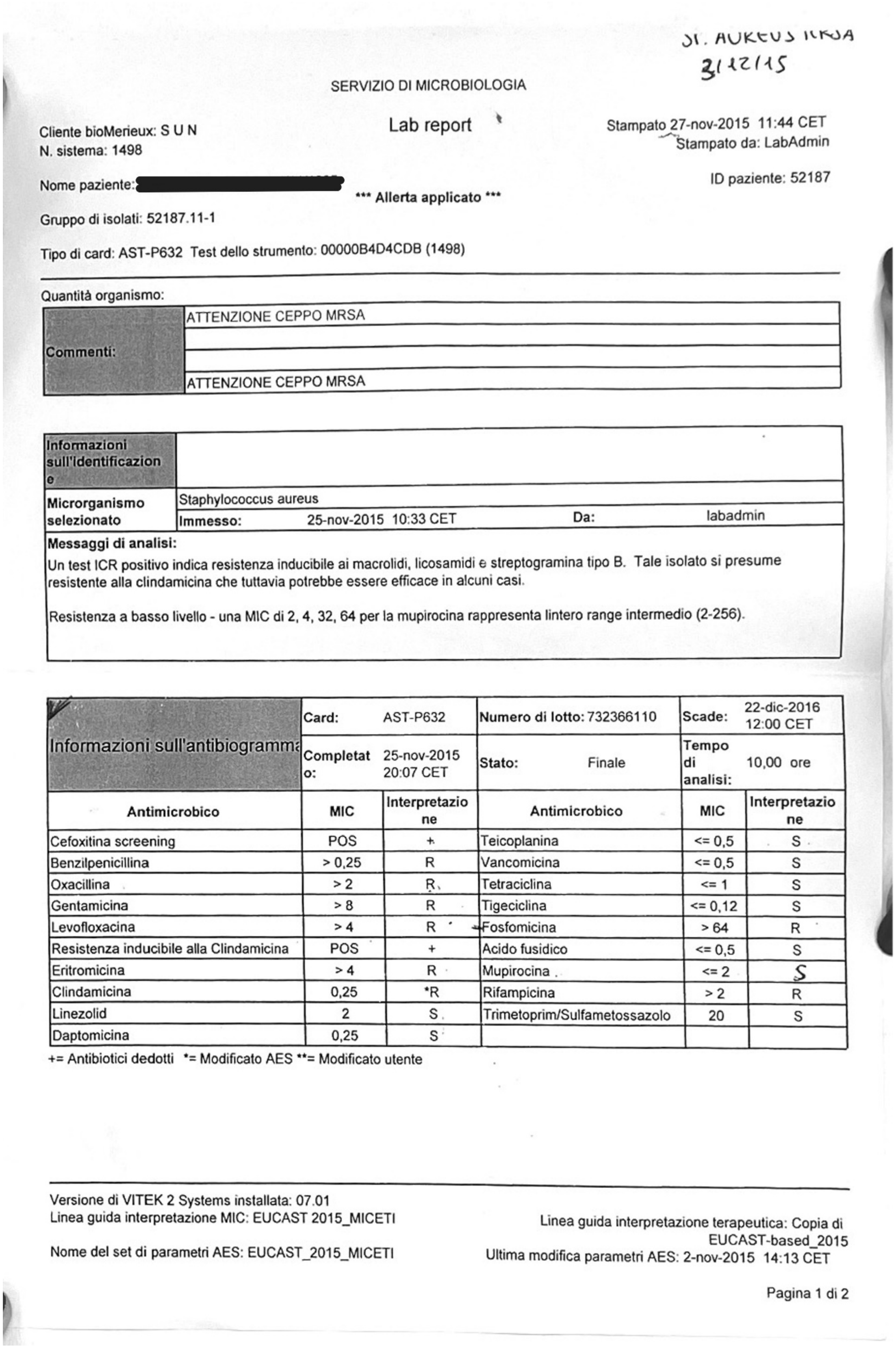

St. AUREUS MRSA
3/12/15

SERVIZIO DI MICROBIOLOGIA

Lab report

Cliente bioMerieux: S U N
N. sistema: 1498

Stampato 27-nov-2015 11:44 CET
Stampato da: LabAdmin

Nome paziente: [redacted]

*** Allerta applicato ***

ID paziente: 52187

Gruppo di isolati: 52187.11-1

Tipo di card: AST-P632 Test dello strumento: 00000B4D4CDB (1498)

Quantità organismo:

Commenti:	ATTENZIONE CEPPO MRSA
	ATTENZIONE CEPPO MRSA

Informazioni sull'identificazione				
Microrganismo selezionato	Staphylococcus aureus			
	Immesso:	25-nov-2015 10:33 CET	Da:	labadmin

Messaggi di analisi:
Un test ICR positivo indica resistenza inducibile ai macrolidi, licosamidi e streptogramina tipo B. Tale isolato si presume resistente alla clindamicina che tuttavia potrebbe essere efficace in alcuni casi.

Resistenza a basso livello - una MIC di 2, 4, 32, 64 per la mupirocina rappresenta lintero range intermedio (2-256).

Informazioni sull'antibiogramma	Card:	AST-P632	Numero di lotto: 732366110	Scade:	22-dic-2016 12:00 CET
	Completato:	25-nov-2015 20:07 CET	Stato: Finale	Tempo di analisi:	10,00 ore

Antimicrobico	MIC	Interpretazione	Antimicrobico	MIC	Interpretazione
Cefoxitina screening	POS	+	Teicoplanina	<= 0,5	S
Benzilpenicillina	> 0,25	R	Vancomicina	<= 0,5	S
Oxacillina	> 2	R	Tetraciclina	<= 1	S
Gentamicina	> 8	R	Tigeciclina	<= 0,12	S
Levofloxacina	> 4	R	Fosfomicina	> 64	R
Resistenza inducibile alla Clindamicina	POS	+	Acido fusidico	<= 0,5	S
Eritromicina	> 4	R	Mupirocina	<= 2	S
Clindamicina	0,25	*R	Rifampicina	> 2	R
Linezolid	2	S	Trimetoprim/Sulfametossazolo	20	S
Daptomicina	0,25	S			

+= Antibiotici dedotti *= Modificato AES **= Modificato utente

Versione di VITEK 2 Systems installata: 07.01
Linea guida interpretazione MIC: EUCAST 2015_MICETI

Nome del set di parametri AES: EUCAST_2015_MICETI

Linea guida interpretazione terapeutica: Copia di EUCAST-based_2015
Ultima modifica parametri AES: 2-nov-2015 14:13 CET

Pagina 1 di 2

Figure S6. MRSA strain antibiogram.

[illegible]
NOS RO
13/12/15
S. AUREUS
MSSA

A.O.U. SUN - .S.C. BATTERIOLOGIA CLINICA

Cliente bioMerieux:
N. sistema:

Lab report

Stampato 18-nov-2014 11:07 CST
Stampato da: labadmin

Nome paziente [redacted] 24/3/92

*** Allerta applicato ***

ID paziente: 50574

Gruppo di isolati: 50574 11-1

Organismo selezionato: Staphylococcus aureus

**Commenti:**	ESEGUIRE TEST CEFINASI

**Informazioni sull'identificazione**	
**Microrganismo selezionato**	Staphylococcus aureus
	**Immesso:** 7-nov-2014 11:46 CST **Da:** labadmin
**Messaggi di analisi:**	

Informazioni sull'antibiogramma	Card: AST-P632		Numero di lotto: 732323640	Scade: 24-ott-2015 13:00 CDT	
	Completato: 7-nov-2014 20:08 CST		Stato: Finale	Tempo di analisi: 8,50 ore	
**Antimicrobico**	**MIC**	**Interpretazione**	**Antimicrobico**	**MIC**	**Interpretazione**
Cefoxitina screening	NEG	-	Teicoplanina	<= 0,5	S
Benzilpenicillina	0,06	S	Vancomicina	<= 0,5	S
Oxacillina	<= 0,25	S	Tetraciclina	<= 1	S
Gentamicina	<= 0,5	S	Tigeciclina	<= 0,12	S
Levofloxacina	<= 0,12	S	Fosfomicina		
Resistenza inducibile alla Clindamicina	NEG	-	Acido fusidico	<= 0,5	S
Eritromicina	0,5	S	Mupirocina	<= 2	
Clindamicina	0,25	S	Rifampicina	<= 0,03	S
Linezolid	2	S	Trimetoprim/Sulfametossazolo	<= 10	S
Daptomicina	0,5	S			

+= Antibiotici dedotti *= Modificato AES **= Modificato utente

**Conclusioni AES:**		**Ultima modifica:** 8-apr-2014 09:18 CDT	**Set di parametri:** EUCAST2014+MICETI vers. 5.04
**Livello di affidabilità:**	Coerente		
**Fenotipo:**	MACROLIDI/LINCOSAMIDI/STREPTOGRAMINE	RESISTENTE ALLE STREPTOGRAMINE (SGA-SGB)	

Azione	Nome (ID utente)	Data/Ora	Commento
Revisionato da:	(labadmin)	10-nov-2014 09:08 CST	

Versione di VITEK 2 Systems installata: 05.04
Linea guida interpretazione MIC: EUCAST_MOD_2014 Linea guida interpretazione terapeutica: EUCAST-based_MOD_2014
Nome del set di parametri AES: EUCAST2014+MICETIvers. 5.04 Ultima modifica parametri AES: 8-apr-2014 09:18 CDT

Pagina 1 di 1

Figure S7. MSSA strain antibiogram.

st. aureus β
3/12/15

Cliente bioMerieux:
N. sistema:

Lab report

Stampato 18-nov-2014 11:06 CST
Stampato da: labadmin

Nome paziente: [redacted]31/3/89
Gruppo di isolati: 52279-1

ID paziente: 52279.10

Organismo selezionato: Staphylococcus aureus

**Commenti:**	

**Informazioni sull'identificazione**		
**Microrganismo selezionato**	Staphylococcus aureus	
	**Immesso:** 23-ott-2014 11:19 CDT	**Da:** labadmin
**Messaggi di analisi:**		

Informazioni sull'antibiogramma					
	**Card:** AST-P632		**Numero di lotto:** 732323640	**Scade:**	24-ott-2015 13:00 CDT
	**Completato:** 23-ott-2014 17:48 CDT		**Stato:** Finale	**Tempo di analisi:**	8,25 ore
**Antimicrobico**	**MIC**	**Interpretazione**	**Antimicrobico**	**MIC**	**Interpretazione**
Cefoxitina screening	NEG	-	Teicoplanina	<= 0,5	S
Benzilpenicillina	>= 0,5	R	Vancomicina	1	S
Oxacillina	0,5	S	Tetraciclina	<= 1	S
Gentamicina	4	R	Tigeciclina	<= 0,12	S
Levofloxacina	0,25	S	Fosfomicina		
Resistenza inducibile alla Clindamicina	NEG	-	Acido fusidico	<= 0,5	S
Eritromicina	>= 8	R	Mupirocina	<= 2	
Clindamicina	0,25	S	Rifampicina	<= 0,03	S
Linezolid	2	S	Trimetoprim/Sulfametossazolo	<= 10	S
Daptomicina	1	S			

+= Antibiotici dedotti *= Modificato AES **= Modificato utente

Conclusioni AES:	**Ultima modifica:** 8-apr-2014 09:18 CDT	**Set di parametri:** EUCAST2014+MICETIvers. 5.04
**Livello di affidabilità:**	Coerente	

Azione	Nome (ID utente)	Data/Ora	Commento
**Revisionato da:**	**(labadmin)**	**3-nov-2014 12:13 CST**	

Versione di VITEK 2 Systems installata: 05.04
Linea guida interpretazione MIC: EUCAST_MOD_2014 Linea guida interpretazione terapeutica: EUCAST-based_MOD_2014
Nome del set di parametri AES: EUCAST2014+MICETIvers. 5.04 Ultima modifica parametri AES: 8-apr-2014 09:18 CDT

Pagina 1 di 1

Figure S8. β-lactamase producer strain antibiogram.

REFERTO - FINALE

Pagina 1/1
26/07/2017 10:08:46

Nome paziente: [redacted] **ID paziente:51883.07**
Data di nascita: Sesso paziente:Non specificato

N accesso: **51883.07**
Tipo campione: CATETERE VENOSO CENTRALE
Reparto ospedaliero: 7301 TER INTENSIVA NEONATALE
Data ricezione: 21/07/2017 09:49:51

Nome organismo **Commenti**
1 STAAUE Staphylococcus aureus

Marker di resistenza
1 STAIML Fenotipo MLSb inducibile di Staphylococcus

**Antibiotico**	**STAAUE** MIC/*Conc.*	SIR
Acido fusidico	<=0,5	S
Ampicillina		R
Ceftaroline	0,5	S
Ciprofloxacina	>4	R
Clindamicina		R
Daptomicina	<=0,5	S
Eritromicina	>2	R
Fosfomicina c/G6P	<=16	S
Gentamicina	2	R
Linezolid	2	S
Moxifloxacina	>1	R
Mupirocina alto livello	<=256	S
Oxacillina	0,5	S
Penicillina G	>0,25	R
Teicoplanina	<=0,5	S
Tetraciclina	<=0,5	S
Tigeciclina	<=0,25	S
Trimetoprim-sulfametoxazolo	<=1/19	S
Vancomicina	1	S

Figure S9. QRSA strain antibiogram.

3/12/15

Cliente bioMerieux. Rapporto grafico Microbiologia Stampato [illegible]

Nome paziente: [redacted]
Posizione:
ID lab.: 51119

ID paziente:
Medico:
Numero di isolato: 1

Organismo selezionato: Staphylococcus aureus

Prelevato:

Origine:

Commenti:

Informazioni sull'antibiogramma	Tempo di analisi:	9,50 ore		Stato:	Finale
**Antimicrobico**	**MIC**	**Interpretazione**	**Antimicrobico**	**MIC**	**Interpretazione**
Cefoxitina screening	NEG	-	Daptomicina	1	S
Benzilpenicillina	>= 0,5	R	Teicoplanina	<= 0,5	S
Oxacillina	<= 0,25	S	Vancomicina	<= 0,5	S
Gentamicina	<= 0,5	S	Tetraciclina	<= 1	S
Levofloxacina	0,25	S	Tigeciclina	<= 0,12	S
* Resistenza inducibile alla Clindamicina	NEG	-	Acido fusidico	<= 0,5	S
* Eritromicina	>= 8	R	Rifampicina	<= 0,03	S
Clindamicina	>= 4	R	Trimetoprim/Sulfametossazolo	<= 10	S
Linezolid	2	S			

+= Antibiotici dedotti *= Modificato AES **= Modificato utente

Conclusioni di AES		
**Affidabilità:**	Coerente	
**Fenotipo:**	MACROLIDI/LINCOSAMIDI/STREPTOGRAMINE	MLSB+SA COSTITUTIVO

Figure S10. MLSB strain antibiogram
